# A Retrospective Study of Adult and Pediatric D‐Dimer Tests to Identify Opportunities for Improved Utilization

**DOI:** 10.1111/ijlh.70024

**Published:** 2025-11-14

**Authors:** Rabab Al Dawood, Hassan Abu Sabah, Natalie Mathews, James Douketis, Catherine Hayward

**Affiliations:** ^1^ Department of Pathology and Molecular Medicine McMaster University Hamilton Canada; ^2^ Department of Pathology and Laboratory Medicine King Fahad Specialist Hospital Dammam Saudi Arabia; ^3^ Department of Pathology and Laboratory Medicine King Faisal Medical City for Southern Regions Abha Saudi Arabia; ^4^ Hematology/Oncology, Centre Hospitalier Universitaire Sainte‐Justine Montréal Québec Canada; ^5^ Department of Medicine McMaster University Hamilton Canada

**Keywords:** coagulation, D‐dimer, DIC, fibrinolysis, pediatrics, thrombosis

## Abstract

**Introduction:**

D‐dimers are produced by lysis of cross‐linked fibrin. In children, D‐dimer testing is used to evaluate disseminated intravascular coagulation (DIC) and some inflammatory states, but its use is not validated for screening or ruling out suspected venous thromboembolic events (VTE). In adults, D‐dimers are used to evaluate DIC, and a low D‐dimer level is used to exclude VTE in patients when combined with a low or moderate probability of VTE.

**Methods:**

To assess D‐dimer utilization and opportunities for improvement, we conducted a retrospective, consecutive‐case cohort study (with ethics approval) of patients who had D‐dimer tests at Hamilton hospitals, from 06/2022 to 05/2023.

**Results:**

D‐dimer levels were evaluated for 175 children and 200 adults (respective results, children/adults: median ages: 12/63, ranges 0–17/18–94 years), and an overlapping cohort of 99 consecutive persons with ≥ 5 D‐dimer determinations. Most patients had D‐dimer tests in emergency departments (60%/62%) and elevated D‐dimer levels (55%/61%). Reasons for D‐dimer assessment included: suspected VTE (50%/70%), DIC (17%/3.5%), childhood inflammatory conditions (23%/0%), and “off‐label” uses (e.g., arterial thrombosis assessment; 5%/22%). VTE likelihood and DIC scores were rarely documented. Patients with multiple tests accounted for 15% of D‐dimer workload. Among patients with multiple tests for DIC, most had overt DIC on initial assessment, with declining D‐dimer levels over 10–14 days, including patients who died.

**Conclusion:**

VTE and DIC assessment was the most common reason for D‐dimer assessments for children and adults. Quality improvement initiatives are needed to improve relevant clinical VTE and DIC score documentation and D‐dimer test utilization.

## 1 Introduction

D‐dimer is a soluble degradation product of plasmin‐digested, factor XIII‐cross‐linked fibrin, with a half‐life of approximately 8 h [1, 2]. Baseline levels are higher in pre‐pubescent children, pregnant women, and adults over 50 years of age [1, 2]. An elevated D‐dimer is a marker of increased coagulation and fibrinolysis, which can result from multiple conditions that include venous thromboembolic disease (VTE), comprising deep venous thrombosis (DVT) or pulmonary embolism (PE), disseminated intravascular coagulation (DIC), cancer, acute infections and inflammatory conditions such as systemic juvenile inflammatory arthritis (sJIA), among others [3, 4].

After the prothrombin time (PT)/International Normalized Ratio (INR) and the activated partial thromboplastin time (aPTT), D‐dimer is the most frequently ordered coagulation test [5]. Notably, D‐dimer is an expensive test: our local reagent cost/test for D‐dimer is approximately 105–120 times higher than that of PT/INR and aPTT reagents from the same vendor. As the PT/INR and aPTT have been a focus of Choosing Wisely Campaigns (https://choosingwiselycanada.org/) to reduce wasteful and unnecessary testing [6], we postulated that an assessment of D‐dimer test utilization would be a helpful first step to similarly address D‐dimer tests.

Trends in D‐dimer test utilization in healthcare have not been recently assessed. Much of the literature on D‐dimer testing is focused on the assessment of specific conditions, commonly VTE or DIC. D‐dimer levels below cutoff values, in combination with a low‐to‐moderate clinical probability for VTE, as assessed by validated clinical prediction rules (e.g., Wells score, Geneva score, YEARs algorithm), can help exclude VTE in adults [7, 8, 9, 10, 11, 12]. Nonetheless, there is uncertainty about how commonly these probabilities are documented in patients who have D‐dimer testing for VTE exclusion outside of clinical studies. Additionally, D‐dimer use for VTE exclusion (or diagnosis) is not validated in children, who have a much lower baseline VTE risk than adults [13, 14]. D‐dimer levels have been used to assess adults and children with unprovoked VTE who are at high risk for recurrence [3, 4, 15, 16, 17, 18, 19]. Some research studies have explored “off‐label” D‐dimer test use for assessment of stroke (with the magnitude of D‐dimer elevation correlating with stroke severity and large vessel occlusion) [20, 21, 22], acute aortic syndromes (although imaging remains the diagnostic gold standard) [23], and arteriovenous malformations (AVM), as 40% have elevated D‐dimers unlike other venous lesions [24].

D‐dimer tests are important for the assessment of impending or overt DIC, typically using the International Society on Thrombosis and Haemostasis (ISTH) DIC scoring scheme that incorporates the magnitude of the D‐dimer increase, PT prolongation, fibrinogen level and platelet count, with high ISTH‐DIC scores in adults associated with overt DIC and a worse prognosis, with further scoring 1–2 days later recommended for patients at risk of progression to overt DIC [25, 26, 27]. ISTH‐DIC scores have similarly been used to assess children with DIC [28].

During the COVID‐19 pandemic, D‐dimer levels were used to assess risks for severe COVID‐19 infection and VTE complications [15, 17]. D‐dimer tests have also been used “off‐label” to evaluate: vaccine‐induced thrombotic thrombocytopenia (VITT) and heparin‐induced thrombocytopenia (HIT) [29]; cytokine release syndrome from chimeric antigen receptor (CAR) T‐cell therapy [30]; and childhood inflammatory diseases such as known or suspected multisystem inflammatory syndrome in children (MIS‐C), and Kawasaki disease (KD) and sJIA [31].

Gaps in current information on D‐dimer test utilization, across the age spectrum, led us to initiate this retrospective, consecutive‐case cohort study. Our primary objective was to assess current D‐dimer test utilization for pediatric and adult patients, including the common reasons for D‐dimer evaluation and typical findings, and identify opportunities for improved test utilization. Our secondary objective was to evaluate findings for consecutive patients with multiple (≥ 5) D‐dimer tests, during a period of care, to address repeated testing.

## 2 Materials and Methods

We conducted a retrospective, consecutive case, cohort study, which was approved by the Hamilton Integrated Research Ethics Board (project 16126). Data for consecutive D‐dimer tests, with medical record numbers (MRN), were obtained by a data pull of Hamilton Regional Laboratory Medicine Program (HRLMP) test records for Hamilton Health Science (HHS) and St. Joseph's Healthcare (SJH) hospital sites in Hamilton, for the period between 06/2022 and 02/2023, and between 06/2022 and 05/2023 to accrue sufficient patients with multiple tests. Anonymized patient identifiers were assigned at the time of data extraction.

Electronic medical record (EMR) reviews were conducted to obtain anonymized, detailed information on representative evaluated patients, including: the first 100–200 consecutive pediatric patients and 200 consecutive adult patients who had D‐dimer tests between 06/2022 and 02/2023, and an overlapping cohort of ~100 consecutive patients (all ages) with≥ 5 D‐dimer determinations between 06/2022 and 05/2023. First encounter D‐dimer results were evaluated for patients with multiple encounters. Anonymized data collected included: patient age, sex (male, female); admission status (outpatient or inpatient); admission duration; if alive or deceased on discharge; indication for D‐dimer testing (consensus used to resolve unclear indications); number of D‐dimer tests; VTE pretest probability or ISTH‐DIC scores (if recorded); relevant medical history, including: previous thrombosis, acute thrombosis, HIT or VITT, purpura fulminans, COVID‐19 infection, current malignancy, CAR T‐cell therapy, evidence of severe liver disease (elevated alanine transaminase ≥ 3 times upper reference interval (RI) limit, or total bilirubin ≥ 2 times upper RI limit) or renal impairment (history of chronic renal disease and/or serum creatinine ≥ 2 times upper RI limit), recent surgery (within the prior 50 days before D‐dimer testing), details of anticoagulation therapy, if any, and laboratory findings for: D‐dimers and other relevant coagulation tests, platelet counts, and liver and renal function tests. ISTH‐DIC scores were calculated for cases with suspected DIC, when possible, using the latest recommendations for scoring D‐dimer elevations [25]. D‐dimer tests were only orderable as separate tests. Only CAR T‐cell therapy order sets included daily D‐dimer tests.

### 2.1 D‐Dimer Testing

D‐dimer was measured using the Werfen HemosIL D‐dimer HS 500 (Bedford, MA), run on Werfen ACL TOP Family 50 Series instruments, using common lots of reagents and controls across all sites. The manufacturer's cutoff of 500 μg/L FEU was verified with adult control samples (*n* = 22; all below the Stago Liatest D‐DI assay cutoff). D‐dimer levels of 215–7650 μg/L FEU were read off a single calibration curve (locally validated for linearity with 20 samples), and higher levels were automatically determined after 1:15 dilution with HemosIL Factor diluent to report values up to 128 000 μg/L FEU. For children, published RI for D‐dimer HS 500 [1] (lower limits rounded up to ≤ 215 μg/L FEU), auto‐attached to results, were used to interpret findings.

### 2.2 Study Outcomes and Statical Analyses

Primary outcomes evaluated were: clinical reasons for D‐dimer testing and D‐dimer test findings, for the consecutive patient cohort, including those with ≥ 5 D‐dimer tests, to identify opportunities to improve test utilization.

Descriptive statistics were used to describe test findings and patient characteristics, using counts and frequencies for categorical variables (e.g., sex, reason for D‐dimer testing) and medians and interquartile range estimates (IQR) for continuous variables (e.g., age, D‐dimer levels). D‐dimer results below detection were rounded up to 215 μg/L FEU (lowest reportable value) for quantitative statistics. Differences were assessed using the Chi‐square test for categorical variables and the Mann–Whitney U test for continuous variables.

## 3 Results

**FIGURE 1:**
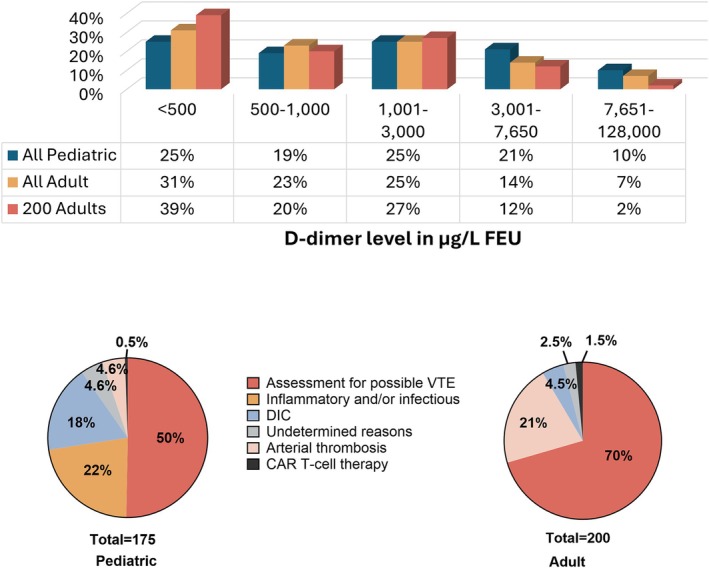
D‐dimer levels for adult and pediatric patients and the reasons for their D‐dimer assessments. The upper panel illustrates the very similar distributions of D‐dimer levels for consecutive adult and pediatric cases, and all adult cases for the 9‐month period evaluated (repeats included). The lower panel pie charts illustrate the reasons for D‐dimer tests among consecutive pediatric and adult patients. Among patients assessed during chimeric antigen receptor (CAR) T‐cell therapy, all were adults, evaluated before and during CAR T‐cell therapy, whereas one pediatric patient who previously received CAR T‐cell therapy had D‐dimer evaluated as part of coagulopathy investigations. DIC, disseminated intravascular coagulation; VTE, venous thromboembolism.

Over 9 months, HRLMP performed 9031 D‐dimer tests. Most samples were quantified without further dilution (93%, 8407/9031) and < 0.2% (18/9031) were above the measurable range (i.e., > 128 000 μg/L FEU) (Figure 1). Most D‐dimer results (96%, 8708/9031) were for adults; accordingly, it took 9 months vs. 1 week of data to accrue 175 pediatric cases and 200 adult consecutive cases. The distribution of D‐dimer levels for the consecutive adult and pediatric cases resembled that of all adults tested over 9 months (Figure 1).

Table 1, Tables S1 and S2 summarize the demographics and other details for the consecutive patients (respective results shown for pediatric/adult cases, unless otherwise specified), who were of diverse ages (respective medians: 12/63 years, range 0–94 years; % males: 51%/48%). Most patients had a single D‐dimer test (78%/94%; median 3 for those with > 1 test between 06/2022 and 02/2023), that showed elevated levels (55%/61%) (Table 1). Most patients (60%/62%) had D‐dimer tests done while in emergency departments, and fewest were tested as outpatients (6.3%/15%) (Table 1). Evaluated patients had a low mortality (1.7%/3.5% deceased on discharge) and few had recent surgery or comorbidities such as severe liver or renal disease (Table 1). Some patients had coagulopathies, most commonly, an elevated PT/INR (Table 1).

**TABLE 1:** Demographics and other details for the patients who had D‐dimer tests.

Characteristics	Results for children	Results for adults
Age in years as median (range, IQR)	12 years (0–17, IQR: 3–15)	63 years (18–94, IQR: 45–73)
Number of males/females, % males	89 male/86 female, 51% male	95 male/105 female, 48% male
% (number) with a single D‐dimer test during an assessment or admission	78% (136)	94% (187)
% (number) with multiple D‐dimer tests during an assessment or admission	22% (*n* = 39) (median 3, range 2–21 tests/patient)	6.5% (*n* = 13) (median 3, range 2–23 tests/patient)
% (number) evaluated by location	Emergency: 60% (*n* = 105; 47% admitted)	Emergency: 62% (*n* = 167; 28% admitted)
	Inpatients: 34% (*n* = 59; PICU: 39%; medical wards: 34%; NICU: 17%; surgical wards: 10%)	Inpatients: 23% (*n* = 62; ICU: 11%; medical wards: 10%; surgical wards: 2%)
	Outpatients: 6.3% (*n* = 11)	Outpatients: 15% (*n* = 41; ODS: 12.5%; Clinics: 2.5%)
Length of stay in days for admitted patients (median, range, IQR)	7 days (1–189, IQR 3–15)	12 days (2–176, IQR 6–24.5)
% (number) deceased at discharge	1.7% (*n* = 3, all with elevated D‐dimer)	3.5% (*n* = 7, not all of them with elevated D‐dimer)
% (number) with recent surgery	7.4% (*n* = 13)	9% (*n* = 18)
% with severe liver disease	8% (*n* = 14)	6% (*n* = 12)
% with severe kidney disease	7.4% (*n* = 13)	18% (*n* = 35)
% with elevated D‐dimer, *n* = 175	55% (*n* = 96/175)	61% (*n* = 122/200)
	> 3000: 34% (*n* = 33/96)	> 3000–7000: 19% (*n* = 23/122)
	> 7000: 17% (*n* = 16/96)	> 7000: 4.9% (*n* = 6/122)
% with elevated D‐dimer above the VTE cut‐off among patients assessed for VTE	39% (*n* = 34/88)	64% (*n* = 90/140)
% with elevated PT among patients with PT tests	46% (*n* = 59/128)	53% (*n* = 59/111)
% with elevated aPTT among patients with aPTT tests	11% (*n* = 11/100)	21% (*n* = 8/38)
% with low fibrinogen among patients with fibrinogen tests	6.4% (6/94)	14% (3/21)
% with prolonged PT and/or aPTT, and/or low fibrinogen among patients who had all three tests	59% (51^a^/86)	83% (15/18)
% with thrombocytopenia	25% (43/169)	18% (34/193)

Abbreviations: aPTT, activated partial thromboplastin time; FEU, fibrinogen equivalent unit; IQR, interquartile range; *n*, number; NICU, neonatal intensive care unit; PICU, pediatric intensive care unit; PT, prothrombin time.

^a^ The 51 includes six patients whose only abnormality was a mildly prolonged PT (INR values were within the reference range).

### 3.1 Reasons for D‐Dimer Tests and Findings

The lower panel of Figure 1 summarizes the reasons for D‐dimer tests among the consecutive adult and pediatric patients.

Most children had D‐dimer testing to assess suspected or known VTE (50%, 88/175, *n* = 31 ≥ 16 years old), followed by inflammatory and/or infectious conditions (22%, 39/175), DIC (18%, 31/175), arterial thrombosis (4.6%, 8/175), undocumented/undetermined reasons (4.6%, 8/173) and one child who was post‐CAR T‐cell therapy had D‐dimer tests as part of coagulopathy investigations (0.6%, 1/175). Children with D‐dimer evaluation for VTE assessment were significantly older than the children assessed for inflammatory and/or infectious conditions (respective median age [IQR, range] in years: 15 [12–16; range: 1–17] vs. 4 [1–10; range: 0–16]; *p* < 0.01).

**FIGURE 2:**
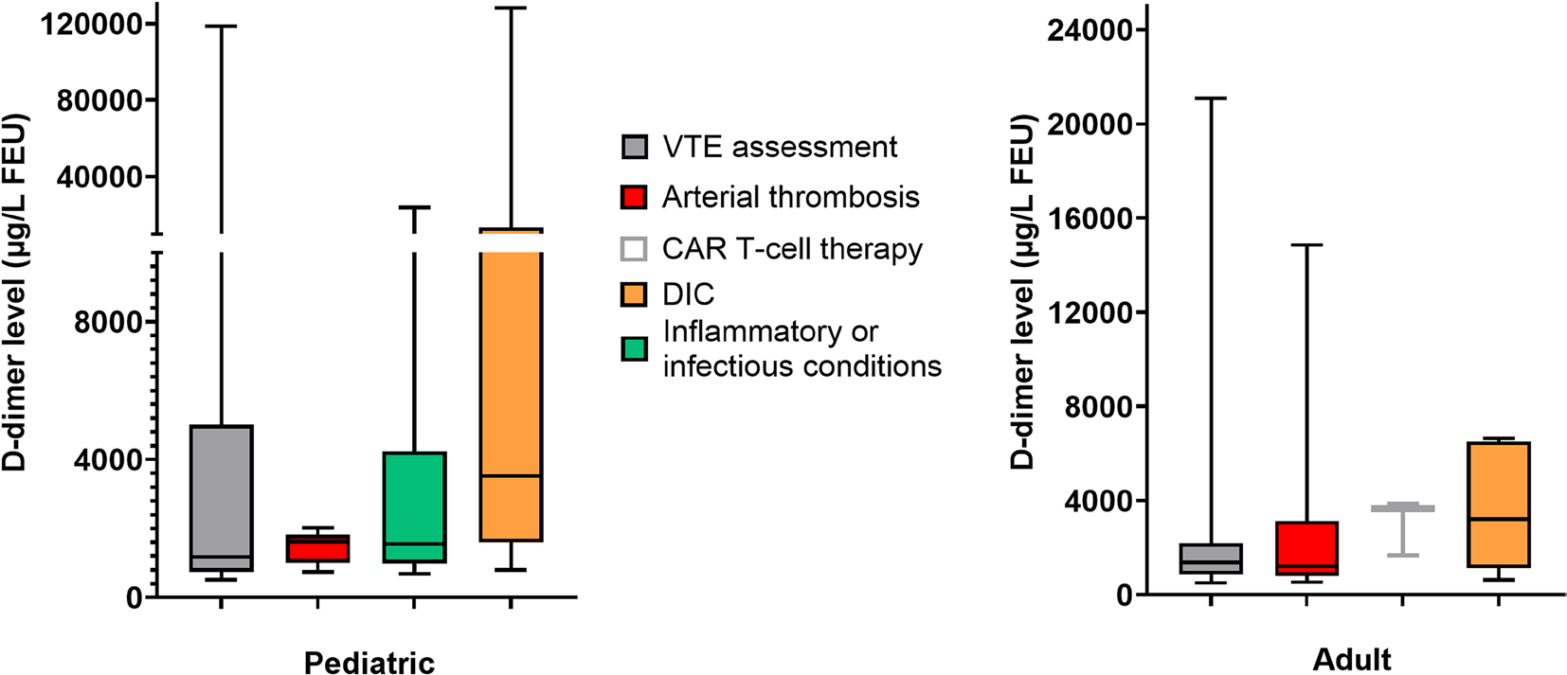
Distribution of D‐dimer levels above the cutoff among consecutive pediatric and adult patients who had D‐dimer assessed for different reasons. First D‐dimer results during the period of evaluation are shown. Results shown were above age‐specific cutoffs for children and above the validated cut‐off of ≥ 500 μg/L for adults. There was a wide range of abnormal findings, and overlap in D‐dimer levels, for the groups evaluated, with whiskers indicating the minimum and maximum values and boxes indicating the interquartile range and median values. CAR, chimeric antigen receptor; DIC, disseminated intravascular coagulation; VTE, venous thromboembolism.

Most adults had D‐dimer testing for VTE (70%, 141/200) or arterial thrombosis (21%, 42/200), and less commonly for: DIC (4.5%, 9/200); acute CAR‐T‐cell therapy monitoring (1.5%, 3/200); or undocumented/undetermined reasons (2.5%, 5/200). Figure 2 and Table S3 show the wide, overlapping distributions of abnormal D‐dimer results for consecutive cases tested for different reasons, with the highest median D‐dimer levels among patients tested for DIC (*p* < 0.01).

### 3.2 D‐Dimer Findings Among Consecutive Children and Adults Assessed for VTE

Among children with D‐dimer testing for VTE, 39% (34/88) had a D‐dimer level above age‐specified cutoffs (median: 1170 μg/L FEU range 522–119 000 μg/L FEU). Most of these children (89%, 78/88) were assessed for a suspected VTE (PE: 78%, 61/78; DVT: 17%, 13/78; other site thrombosis: 5.1%, 4/78), and others, during anticoagulation therapy for VTE (11%, 10/88). Eight older children (ages: 9–16 years; 3/8 with elevated D‐dimer levels) had documented VTE pretest probability scores (*n* = 3 PERC; *n* = 3 Wells; *n* = 2 both Wells and PERC) that indicated a low‐to‐moderate probability of VTE and the three with elevated D‐dimers underwent imaging (two negative for PE by CTPA, the other positive for lesser saphenous vein thrombosis by Doppler ultrasound). Overall, more children with elevated than normal D‐dimer levels underwent imaging for VTE (20/26 vs. 5/52, *p* < 0.001) that included: CTPA, *n* = 13; Doppler ultrasounds for DVT, *n* = 9; magnetic resonance imaging (MRI) for cerebral venous sinus thrombosis (CSVT), *n* = 1; and abdominal ultrasounds, *n* = 2. Among the entire pediatric cohort, 2 children were diagnosed with new VTE (*n* = 1 PE, *n* = 1 DVT; respective D‐dimers: 827 vs. 31 100 μg/L FEU) and 10 others had D‐dimers assessed while on anticoagulation for diagnosed VTE (80%, 8/10 evaluated < 10 days after a new VTE diagnosis; sites: CVST, *n* = 5; PE, *n* = 1; DVT and PE, *n* = 1; hepatic plus limb DVT, *n* = 1; *n* = 2 tested later on anticoagulation for DVT). Among cases assessed early during anticoagulation, D‐dimers were lower for cases with a recent CVST than other site thromboses (medians: 930 vs. 51 800 μg/L; ranges: 498–7450 vs. 14 100–119 000 μg/L FEU, *p* = 0.025) and highest for the child with antithrombin deficiency and a recent, massive bilateral PE. Among the two children who were tested after more prolonged anticoagulant therapy, one had normal D‐dimer levels (246 μg/L FEU) 1 month into anticoagulation for DVT whereas the other had significantly elevated D‐dimer levels (16 900 μg/L FEU) after > 12 months of continuous anticoagulation therapy for recurrent, unprovoked VTE.

Most adults who had D‐dimer testing for VTE (*n* = 140, including *n* = 2 tested while on anticoagulation for VTE) had levels above the VTE diagnostic cutoff (≥ 500 μg/L FEU) (64%, 90/140; median [range]: 854 [215–21 100] μg/L FEU). The majority had D‐dimer testing for suspected PE (64%, 89/140; 65%, 58/89 with elevated D‐dimer) or DVT (36%, 51/140; 65%, 33/51, with elevated D‐dimer) but very few had documented, pretest probability scores (15%, 21/140; Wells score: *n* = 11; YEARS criteria: *n* = 5; Wells and PERC scores: *n* = 3; Wells and YEARS criteria: *n* = 2). Most adults who underwent imaging for suspected VTE had elevated D‐dimer levels (86%, 62/72), with a thrombosis detected in 18% (11/62, *n* = 8 PE; *n* = 3 DVT; 10/11 with elevated D‐dimer levels, none with a documented pretest probability score). Only two adults had D‐dimer assessed during anticoagulation for recent VTE (one normal, the other mildly increased at 522 μg/L FEU).

### 3.3 D‐Dimer Findings for Children and Adults Assessed for Arterial Thrombosis

Among children with D‐dimer tests for a possible arterial thrombosis (*n* = 5 evaluated for stroke, *n* = 3 for extremity thrombosis), there were no significant differences in the D‐dimer levels among those who had (*n* = 3 stroke on MRI; *n* = 2 arterial limb thrombosis, one post‐traumatic treated by angioplasty) or did not have an arterial thrombosis (*n* = 5) (respective μg/L FEU: medians: 1290 vs. 302; ranges: 644–2020 vs., < 215–1630; *p* = 0.25). Similarly, among adults with D‐dimer tests for arterial thrombosis (93% 39/42 with elevated D‐dimer levels) there were no significant differences in D‐dimer levels among those who had (*n* = 4 with non‐ST‐elevation myocardial infarction; *n* = 1 ST‐elevation myocardial infarction; *n* = 1 stroke; *n* = 1 limb ischemia from iliac artery thrombosis) or did not have an arterial thrombosis (*n* = 35) (respective μg/L FEU: medians: 371 vs. 388; ranges 296–14 900 vs. < 215–4640; *p* = 0.34).

### 3.4 D‐Dimer Results for Children Assessed for Inflammatory and Infection Conditions

Only children (23%, 40/175) in the cohort had D‐dimer tests to assess inflammatory and/or infectious conditions, including: query KD or MIS‐C (*n* = 20); known or suspected COVID‐19 infection (*n* = 16); systemic juvenile idiopathic arthritis (sJIA, *n* = 3 tested to assess disease severity, as recommended) [31]; and another had D‐dimer tests as part of coagulopathy investigations post CAR T‐cell therapy (D‐dimer level normal). D‐dimer levels for these patients varied (range: < 215–24 000 μg/L FEU; elevated in 82.5%, 33/40) and were highest for a child with a sJIA flare [24 000 μg/L FEU] and a child with COVID‐19 complicating Mabry syndrome and pneumatosis [16 200 μg/L FEU]. Most children with suspected KD or MIS‐C had mild to modestly elevated D‐dimer levels (85%, 17/20; range: 820–6540 μg/L FEU; all ≤ 10 years of age; two requiring ICU care; *n* = 6 ultimately diagnosed with KD [*n* = 3], MIS‐C [*n* = 1] or both [*n* = 2], treated with aspirin). Most children tested because of suspected or confirmed COVID‐19, requiring hospitalization, had confirmed COVID‐19 (81%, 13/16, all requiring respiratory support, none with acute thrombosis or COVID‐19‐associated coagulopathy) often with elevated D‐dimers (77%, 10/13, range: 889–16 200 μg/L FEU; nine requiring ICU care; *n* = 2 given LMWH, one for thromboprophylaxis, the other given therapeutic LMWH for a portal vein thrombosis, 4 months earlier).

### 3.5 D‐Dimer Findings for Children and Adults Evaluated for DIC

Thirty‐one children (18% of all children) had D‐dimer tests for assessment of systemic DIC (87%, 27/31; 21/27 with thrombocytopenia; 20/27 with elevated D‐dimer levels) or localized DIC from a venous malformation (*n* = 4; 2/4 with elevated D‐dimer levels). Among children assessed for systemic DIC who had elevated D‐dimer levels, only 26% (7/27) had overt DIC by ISTH DIC scores (*n* = 6 from severe infections, *n* = 1 post‐cardiac arrest) and 18% (5/27, 2 with overt DIC) had an acute thrombosis (DVT, *n* = 3; CVST, *n* = 1; stroke, *n* = 1; D‐dimers elevated in all except the child with stroke).

Among the 9/200 consecutive adults who had D‐dimer tests for DIC assessment (levels, μg/L FEU: median: 1320, range: 328–6600, elevated in 7/9), none had a documented ISTH‐DIC score, and retrospectively, only one scored for overt DIC (score = 6). Additional patients with overt DIC were identified among patients with ≥ 5 D‐dimer tests (described later).

### 3.6 D‐Dimer for Patients Receiving CAR T‐Cell Therapy

Three of the 200 (1.5%) consecutive adult patients, and no children, had D‐dimer assessments during CAR T‐cell therapy, with additional cases in the group with ≥ 5 D‐dimer tests (described later), as the use of CAR T‐cell therapy expanded over the year evaluated.

### 3.7 D‐Dimer Findings for Children and Adults With Poorly Documented Reasons for Testing

Eight children and six adults had D‐dimer tests for undocumented/undetermined reasons (normal in 6/8 children, including 4/5 testing during bleeding disorder investigations; abnormal in 6/6 adults, including the hemophiliac tested at a follow‐up visit; details in Tables S1 and S2).

### 3.8 D‐Dimer Findings for Children and Adults With Five or More D‐Dimer Tests

**FIGURE 3:**
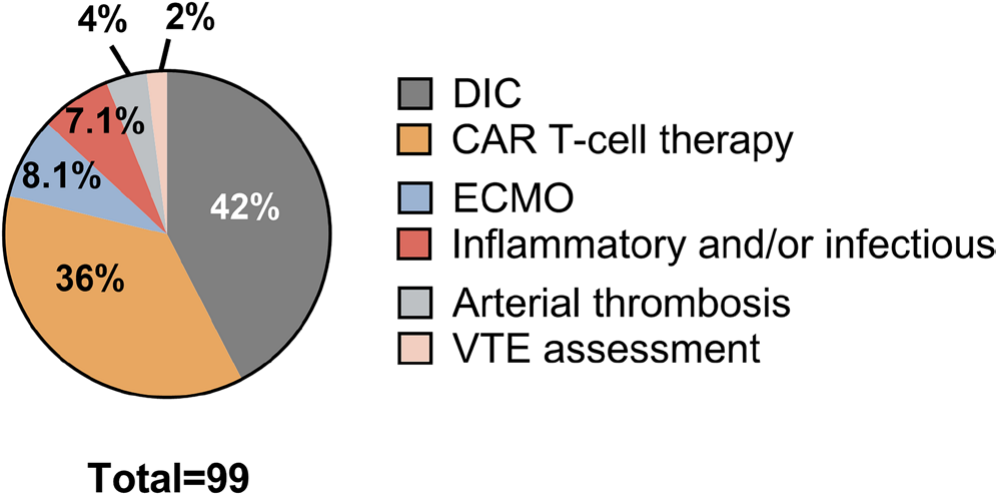
Reasons for D‐dimer tests among consecutive patients who had five or more D‐dimer tests over a period of care. The percentages tested for different reasons are shown. CAR, chimeric antigen receptor; DIC, disseminated intravascular coagulation; ECMO, extracorporeal membrane oxygenation; VTE, venous thromboembolism.

**FIGURE 4:**
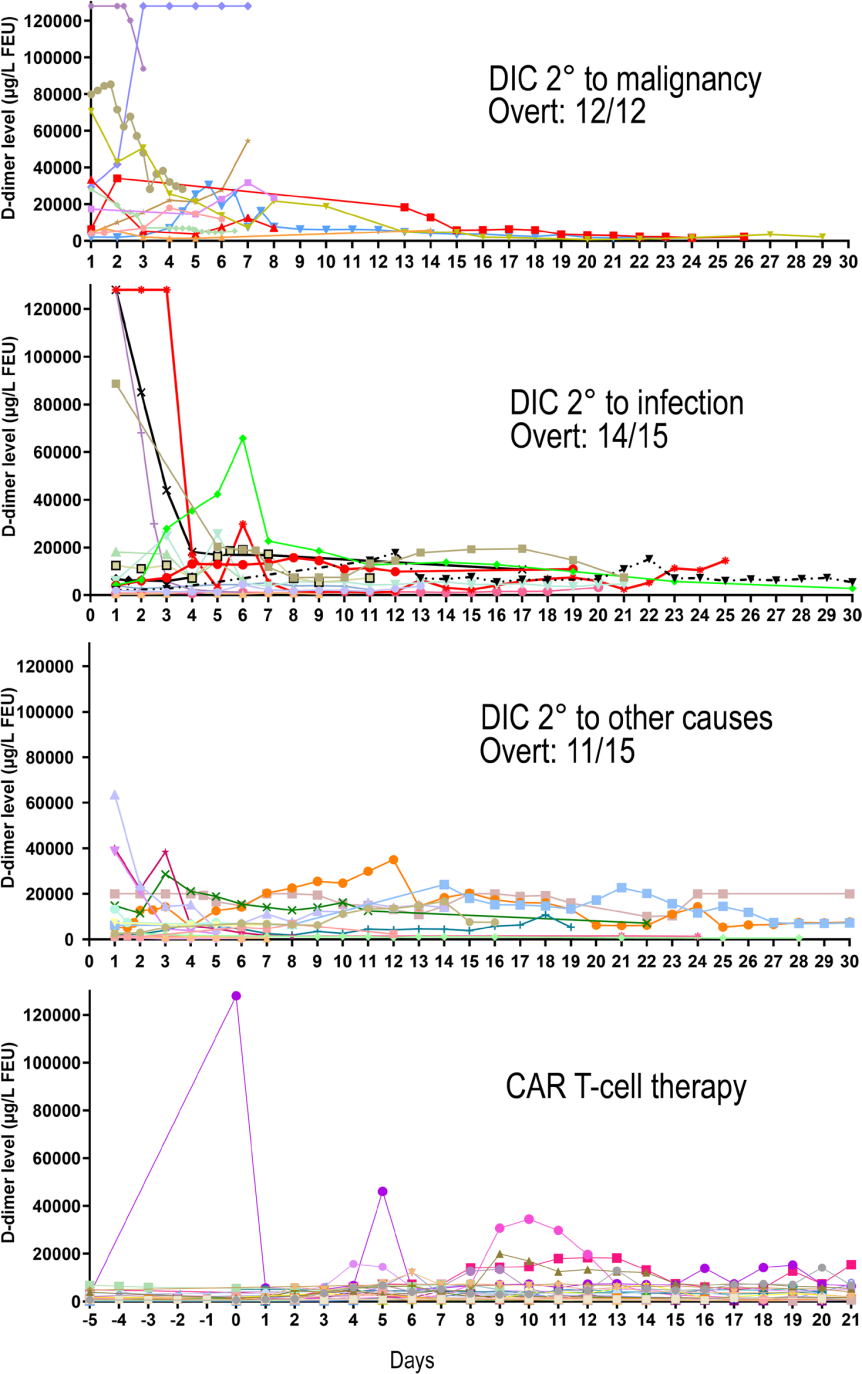
Temporal changes in D‐dimer levels among patients who had five or more D‐dimer levels to evaluate for DIC secondary to malignancy, infection or other causes, or to monitor CAR T‐cell therapy. Panels show daily changes. Patients who were deceased at discharge are shown with red or black lines and symbols. Patients undergoing CAR T‐cell therapy were routinely monitored for 5 days pre‐infusion and for 3 weeks post‐infusion.

Over the year evaluated, 99 consecutive patients (90% adult; 14/99 overlapped the 9 month cohort) were identified with ≥ 5 or more D‐dimer tests over a period of care (details in Table S4); their tests accounted for 15% of the total D‐dimer test workload that year. Figure 3 summarizes their reasons for D‐dimer evaluation, which included: DIC assessment (42%; related to infections [36%], malignancies [29%] or other causes [35%]); monitoring of CAR T‐cell therapy (36%; routinely done between days −5 and 21 post infusion) and less commonly: ECMO (8%); pediatric inflammatory conditions (4%); COVID‐19 infection (3%); arterial thrombosis (3%); or VTE (2%). Figure 4 illustrates the temporal changes in D‐dimer among patients with multiple tests to assess DIC or CAR T‐cell therapy; some were tested 2–4 times within a day, for unclear reasons. Among patients assessed for DIC, the majority scored as overt DIC on first assessment (86%, 36/42) and only one of the others (17%, 1/6) progressed to an overt DIC score due to a decline in platelet count 10 days after developing a post‐bone marrow transplantation coagulopathy, without changes in other DIC score parameters. D‐dimer levels generally declined over time, even among patients with DIC who died (shown in red and black in Figure 4), including the two who had overt DIC from an infection and initial D‐dimer levels ≥ 128 000 μg/L FEU that rapidly declined with anticoagulation. Some physicians discontinued D‐dimer monitoring after diagnosing DIC, whereas others continued to monitor regularly (often daily), even beyond 2 weeks when most DIC patients' D‐dimer levels had plateaued or declined (Figure 4). One case with DIC from infection had a secondary spike in D‐dimer (Figure 4), with imaging ruling out an acute VTE. CAR T‐cell therapy patients had minor fluctuations in D‐dimer levels that did not trigger changes in treatment, raising questions about the value of the routine D‐dimer monitoring.

## 4 Discussion

D‐dimer is a frequently ordered coagulation test that is used to evaluate and rule out certain conditions. To gain insights on D‐dimer test utilization and findings, we retrospectively assessed 175 consecutive children and 200 consecutive adults, and an overlapping cohort of 99 patients (90% adult) with≥ 5 D‐dimer determinations during a single period of evaluation. A major finding of our study is that D‐dimer tests are more commonly utilized to assess adults than children; however, among both, VTE and DIC assessments are two main reasons for D‐dimer testing, with assessments for pediatric inflammatory and/or infection conditions contributing to D‐dimer utilization for children. Strengths of our study include the information on D‐dimer utilization for patients of diverse ages, across multiple sites, and the high potential for external validity given that consecutive case evaluations limited bias. The weaknesses relate to the retrospective study design, which made it difficult to assess how D‐dimer findings influenced patient care. An important observation was the very poor documentation of VTE and ISTH‐DIC scores among patients with D‐dimer tests for VTE and DIC assessment, which limited the characterization of evaluated patients, and should be addressed by quality improvement initiatives.

Patients with ≥ 5 D‐dimer tests accounted for 15% of the annual D‐dimer test workload and were predominantly patients assessed for DIC, or during CAR T‐cell therapy. Choosing Wisely initiative approaches would be helpful to limit wastage in D‐dimer testing. For example, some patients with D‐dimer tests for DIC had 2–4 determinations within 24 h, and often daily for up to a month. We noted that 86% of patients with multiple tests for DIC had scores of overt DIC on initial assessment, with only one additional patient progressing to an overt DIC score after 10 days of follow‐up testing. Experts recommend reassessing for progression to overt DIC 1 or 2 days later [26]. The frequent (often daily) monitoring of D‐dimer levels for patients already diagnosed with overt DIC needs consideration of temporal changes: while some who started treatment of the underlying cause of their DIC or anticoagulation, had a rapid fall in D‐dimer within the first few days and first week of treatment, similar declines, over 10–14 days, were seen among others, including among the patients who died. These observations indicate that daily D‐dimer monitoring of such patients is largely non‐informative. It would be helpful to have more evidence on the prognostic usefulness of ISTH‐DIC scores for children [25, 28] who accounted for 10% of our cases with ≥ 5 D‐dimer tests for DIC.

We were surprised that a growing number of D‐dimer tests for adults were being done during CAR T‐cell therapy, which related to the expansion of that treatment program and the standardized order sets that included daily D‐dimer testing for 26 days. Most CAR T‐cell treated patients showed relatively minor changes in D‐dimer levels, suggesting that CAR T‐cell therapy monitoring may be another condition/scenario that warrants a reduced frequency or discontinuation of D‐dimer assessment. Our study findings do not support D‐dimer testing for suspected arterial thrombosis as the D‐dimer levels for adults and children who had or did not have arterial thrombosis were not significantly different.

Some uses of D‐dimer tests are unique to children: the utilization of D‐dimer tests for children in our study with suspected or known infectious and/or inflammatory conditions was largely in keeping with published recommendations [16, 32]. KD and MIS‐C are well‐documented causes of an elevated D‐dimer level, with MIS‐C cases often having higher levels [32]; we had insufficient cases to verify the latter. The local use of D‐dimer tests for assessment of sJIA and COVID‐19 infection largely aligned with published evidence on use of D‐dimers as a biomarker of disease activity and severity for these conditions [16, 31]. Thromboembolic complications are more commonly reported in adults with COVID‐19 infection compared to children [33]; the longer period of accrual for our pediatric cases (9 months vs. 1 week) is probably why more children than adults were evaluated for COVID‐19 infection.

The incidence of VTE in children is much lower than in adults [34] and there are uncertainties about the use of D‐dimer in VTE exclusion, along with other pre‐test probability scores, for pediatric populations, given the lower risk of VTE [13, 14]. Despite conducting EMR reviews for quite a few children with D‐dimer tests for VTE, we remain uncertain about how D‐dimer results were used for care. This is an important issue as 50% of all D‐dimer requests for children are for VTE assessments. Our study highlights the low prevalence of thrombosis among children tested for D‐dimers with very few children having documented thrombosis and many children with elevated D‐dimers having undergone imaging studies, making it challenging to recommend quality improvement strategies.

We propose the following measures to optimize D‐dimer utilization:

1. Limit overutilization of D‐dimer tests, which are the third most frequently ordered coagulation tests performed in medical laboratories [5, 35, 36]. We suggest that D‐dimer orders should be limited to once per 24 h [4], which is in keeping with recommendations to repeat the test 24 or 48 h later for patients suspected to evolving to overt DIC [26]. The monitoring of D‐dimer levels for DIC more frequently than 2–3 times/week, and beyond 10–14 days, should be discouraged given that D‐dimer levels decline over time among patients with overt DIC, even among those who do not survive. Furthermore, progression to overt DIC beyond 10 days was not observed. D‐dimer testing for “off‐label” purposes (e.g., arterial thrombosis assessment, which our findings do not support) should be discouraged.
2. Enhance documentation of VTE and DIC scores through support tools, communications and educational initiatives. Documentation is a critical component of patient care [37], and we noted suboptimal documentation of both ISTH DIC and VTE risk scores, despite guideline recommendations to use D‐dimer tests in conjunction with scoring systems for both DIC and VTE [7, 25]. We created a template to aid documentation of ISTH DIC scores, and suggest similar measures for VTE risk assessment, to improve the documentation of evidence‐based clinical decision making [37, 38].
3. Advocate for the development of pediatric‐specific guideline recommendations on the appropriate uses of D‐dimer tests for DIC and VTE, and the validity of DIC and VTE scoring schemes for children, to optimize pediatric D‐dimer test utilization.
4. Revisit the routine practice of daily D‐dimer assessment before and for 3 weeks after CAR T‐cell therapy. While these patients are at risk of developing cytokine storm and a coagulopathy with elevated D‐dimers, D‐dimer findings are not an actionable parameter. We suggest that routine D‐dimer assessment during CAR T‐cell therapy should be eliminated or reduced to several times per week.

## Author Contributions

R.A.D. and H.A.S. performed EMR reviews to obtain data and led the manuscript preparation, supervised by C.H., with contributions from all authors. R.A.D. performed statistical analysis. R.A.D. and H.A.S. prepared figures. J.D. and C.H. supervised EMR reviews. C.H. obtained research ethics approval and supervised the study.

## Ethics Statement

This study was approved by the Hamilton Integrated Research Ethics Board (HIREB project 16126).

## Consent

The Hamilton Integrated Research Ethics Board did not require the investigators to obtain patient consent for this retrospective study.

## Conflicts of Interest

The authors have no conflicts of interest to declare for this study. C.H. has received honorariums from Werfen and Stago and royalties from UpToDate, unrelated to this study.

## Supporting information


Data S1.

## Data Availability

The data that supports the findings of this study is available in the Supporting Information of this article.
