# Supplementary material for: A Retrospective Study of Adult and Pediatric D‐Dimer Tests to Identify Opportunities for Improved Utilization
Source: Int J Lab Hematol. 2025 Nov 14;48(2):392–401. doi: 10.1111/ijlh.70024 (PMC12956500; doi:10.1111/ijlh.70024)
Supplement: Supplementary file 1 — Data S1. [file IJLH-48-392-s001.docx]

**Table S1.** **Summary of information on the pediatric patient who had D-dimer tests.** Laboratory results were obtained from historical records, with information on ages and sex. Diagnoses were obtained by review of electronic medical records. Reasons for D-dimer testing (shown below) were coded as follows: (1, deep venous thrombosis; 2, pulmonary embolism; 3, arterial thrombosis; 4, disseminated intravascular coagulation; 5, chimeric antigen receptor T-cell therapy; 6, COVID-19 infection; 7, inflammatory conditions; 8, other venous thromboembolism; 9, other causes; 10, venous thromboembolism assessment; 0, unknown reason). Definitions of abbreviations are located below the table.

| Code | | sex  0=F  1=M | | Age  (yrs) | | collection  department | Alive on discharge 0=no  1=yes | | Reason for  D-dimer testing | | first D-dimer level (µg/FEU) | | D-dimer elevated 1=no,  2=yes | | number  of  D-dimer repeats | | INR | | PT  (sec) | | APTT (sec) | | Fibrinogen  level  (g/L) | | lowest  plt  count (x10^9^/L) | | One or more ISTH DIC scores recorded on chart 0=no  1=yes | | VTE  pretest  probability  score  done  0=no  1=yes  (specify) | | acute  thrombosis  diagnosed  0=no  1=yes  (specify) | | Imaging  done  0=no  1=yes  (specify) | | hepatic impairment  0=no  1=yes | | renal impairment 0=no 1=yes | | recent surgery 0=no 1=yes | | malignancy  0=no  1=yes | |  |
| --- | --- | --- | --- | --- | --- | --- | --- | --- | --- | --- | --- | --- | --- | --- | --- | --- | --- | --- | --- | --- | --- | --- | --- | --- | --- | --- | --- | --- | --- | --- | --- | --- | --- | --- | --- | --- | --- | --- | --- | --- | --- | --- | --- |
| P2586 | 1 | | 1 | | MM PICU | | | 1 | | 2 | | 7570 | | 2 | | 0 | | 1.2 | | 14.3 | | 28 | | 4.2 | | 153 | | 0 | | 0 | | 0 | | 1  (CTPA) | | 0 | | 0 | | 0 | | 0 | |
| P4671 | 0 | | 2 | | MM 3B2 HEM/ONC | | | 1 | | 2 | | 827 | | 2 | | 2 | | 0.9 | | 10.5 | | 25 | | 1 | | 175 | | 0 | | 0 | | 1  (PE) | | 1  (CTPA) | | 0 | | 0 | | 1 | | 1  (B-ALL) | |
| P4245 | 0 | | 4 | | MM EMERGENCY DEPT | | | 1 | | 8 | | 6410 | | 2 | | 0 | | 4.2 | | 49.9 | | 37 | | 2.9 | | 222 | | 0 | | 0 | | 0 | | 1  (abdominal US) | | 1 | | 0 | | 0 | | 0 | |
| P4659 | 0 | | 4 | | MM 3Y1 SURGERY | | | 1 | | 10 | | 7450 | | 2 | | 0 | | 1.1 | | 13 | | 29 | | 6.2 | | 388 | | 0 | | 0 | | 1  (CSVT) | | 1  (head MRI or CT) | | 0 | | 0 | | 1 | | 0 | |
| P6526 | 1 | | 5 | | MM EMERGENCY DEPT | | | 1 | | 10 | | 1100 | | 2 | | 0 | | 1.2 | | 13.3 | | 37 | | 5.3 | | 365 | | 0 | | 0 | | 1  (CSVT/IJV) | | 1  (head MRI or CT) | | 0 | | 0 | | 0 | | 0 | |
| P4246 | 1 | | 5 | | MM PICU | | | 1 | | 10 | | 51800 | | 2 | | 0 | | 1.1 | | 12.5 | | 30 | | 5.9 | | 109 | | 0 | | 0 | | 1 (cephalic/hepatic) | | 1  (doppler US) | | 1 | | 1 | | 1 | | 0 | |
| P6875 | 0 | | 6 | | MM EMERGENCY DEPT | | | 1 | | 2 | | 617 | | 2 | | 1 | | 1.2 | | 13.1 | | NA | | NA | | 252 | | 0 | | 0 | | 0 | | 0 | | 0 | | 0 | | 0 | | 0 | |
| P857 | 1 | | 7 | | MM 3B2 HEM/ONC | | | 1 | | 2 | | 1220 | | 2 | | 1 | | 1.2 | | 13.8 | | 35 | | 3.9 | | 2 | | 0 | | 0 | | 0 | | 1  (CTPA) | | 0 | | 0 | | 0 | | 1  (HL) | |
| P5427 | 1 | | 8 | | MM EMERGENCY DEPT | | | 1 | | 2 | | 320 | | 1 | | 0 | | 1.1 | | 12.3 | | NA | | NA | | 237 | | 0 | | 0 | | 0 | | 0 | | 0 | | 0 | | 0 | | 0 | |
| P9114 | 1 | | 8 | | MM 3B1 EAT DISORDERS | | | 1 | | 10 | | 498 | | 1 | | 0 | | 1.1 | | 12.3 | | 38 | | 5.3 | | 332 | | 0 | | 0 | | 1  (CSVT) | | 1  (head MRI or CT) | | 0 | | 0 | | 0 | | 0 | |
| P1729 | 1 | | 8 | | MM EMERGENCY DEPT | | | 1 | | 10 | | 930 | | 2 | | 0 | | 1.5 | | 16.9 | | 29 | | 7.6 | | 213 | | 0 | | 0 | | 1  (CSVT) | | 1  (head MRI or CT) | | 0 | | 0 | | 0 | | 0 | |
| P6153 | 1 | | 8 | | MM EMERGENCY DEPT | | | 1 | | 2 | | 1250 | | 1 | | 0 | | 1.5 | | 17.2 | | 23 | | NA | | 445 | | 0 | | 0 | | 0 | | 0 | | 0 | | 0 | | 0 | | 0 | |
| P1278 | 0 | | 9 | | MM EMERGENCY DEPT | | | 1 | | 1 | | 31090 | | 2 | | 3 | | 1.2 | | 13.5 | | 28 | | 1.4 | | 210 | | 0 | | 1  (Wells) | | 1  (Lesser saphenous  vein) | | 1  (doppler US) | | 0 | | 0 | | 0 | | 0 | |
| P7230 | 0 | | 9 | | MM 3C MEDICINE | | | 1 | | 2 | | 3520 | | 2 | | 0 | | 1.1 | | 12.7 | | 30 | | NA | | 127 | | 0 | | 0 | | 0 | | 0 | | 0 | | 0 | | 0 | | 0 | |
| P3849 | 1 | | 10 | | MM EMERGENCY DEPT | | | 1 | | 2 | | 308 | | 1 | | 0 | | 1.1 | | 12.8 | | NA | | NA | | 291 | | 0 | | 0 | | 0 | | 0 | | 0 | | 0 | | 0 | | 0 | |
| P478 | 0 | | 12 | | SJHK URGENT CARE | | | 1 | | 1 | | 266 | | 1 | | 0 | | NA | | NA | | NA | | NA | | NA | | 0 | | 0 | | 0 | | 0 | | 0 | | 0 | | 0 | | 0 | |
| P9129 | 1 | | 15 | | MM IR DEP | | | 1 | | 4  (localized) | | 215 | | 1 | | 0 | | NA | | NA | | NA | | 2.5 | | 252 | | 0 | | 0 | | 0 | | 0 | | 0 | | 0 | | 0 | | 0 | |
| P5415 | 1 | | 0 | | MM PICU | | | 1 | | 6 | | 301 | | 1 | | 0 | | 1.2 | | 14.3 | | 36 | | 3 | | 590 | | 0 | | 0 | | 0 | | 0 | | 0 | | 0 | | 0 | | 0 | |
| P8932 | 0 | | 16 | | MM LAB SCC | | | 1 | | 3 | | 215 | | 1 | | 0 | | 1.1 | | 12.5 | | 35 | | 3.4 | | 185 | | 0 | | 0 | | 0 | | 1  (head MRI or CT) | | 0 | | 0 | | 0 | | 0 | |
| P3438 | 0 | | 12 | | MM EMERGENCY DEPT | | | 1 | | 1 | | 390 | | 1 | | 0 | | NA | | NA | | NA | | NA | | 404 | | 0 | | 0 | | 0 | | 0 | | 0 | | 0 | | 0 | | 0 | |
| P3432 | 0 | | 12 | | MM EMERGENCY DEPT | | | 1 | | 1 | | 1120 | | 2 | | 0 | | NA | | NA | | NA | | NA | | 269 | | 0 | | 0 | | 0 | | 1  (doppler US) | | 0 | | 0 | | 0 | | 0 | |
| P5784 | 0 | | 12 | | MM EMERGENCY DEPT | | | 1 | | 2 | | 803 | | 2 | | 0 | | 1.1 | | 12.8 | | NA | | NA | | 338 | | 0 | | 1  (Wells) | | 0 | | 1  (CTPA) | | 0 | | 0 | | 0 | | 0 | |
| P2581 | 1 | | 12 | | MM EMERGENCY DEPT | | | 1 | | 2 | | 215 | | 1 | | 0 | | 1.1 | | 12.8 | | 37 | | NA | | 198 | | 0 | | 1  (PERC) | | 0 | | 0 | | 0 | | 0 | | 0 | | 0 | |
| P3111 | 1 | | 12 | | SJHK URGENT CARE | | | 1 | | 2 | | 324 | | 1 | | 0 | | NA | | NA | | NA | | NA | | 340 | | 0 | | 0 | | 0 | | 0 | | 0 | | 0 | | 0 | | 0 | |
| P6138 | 1 | | 12 | | MM EMERGENCY DEPT | | | 1 | | 2 | | 466 | | 1 | | 0 | | 1 | | 11.7 | | NA | | NA | | 282 | | 0 | | 0 | | 0 | | 0 | | 0 | | 0 | | 0 | | 0 | |
| P5420 | 1 | | 12 | | SJHK URGENT CARE | | | 1 | | 2 | | 1330 | | 2 | | 1 | | 1.4 | | 16 | | 32 | | 7.4 | | 186 | | 0 | | 0 | | 0 | | 1  (CTPA) | | 0 | | 0 | | 0 | | 0 | |
| P5056 | 1 | | 12 | | MM EMERGENCY DEPT | | | 1 | | 8 | | 646 | | 2 | | 0 | | 1.6 | | 18.1 | | 37 | | 5.6 | | 218 | | 0 | | 0 | | 0 | | 1  (head MRI or CT) | | 0 | | 0 | | 1 | | 0 | |
| P5424 | 1 | | 12 | | MM LAB SCC | | | 1 | | 10 | | 16900 | | 2 | | 0 | | NA | | NA | | NA | | NA | | 244 | | 0 | | 0 | | 0 | | 0 | | 0 | | 0 | | 0 | | 0 | |
| P2175 | 0 | | 13 | | MM EMERGENCY DEPT | | | 1 | | 2 | | 215 | | 1 | | 0 | | 1.1 | | 13.1 | | NA | | NA | | 231 | | 0 | | 0 | | 0 | | 0 | | 0 | | 0 | | 0 | | 0 | |
| P4665 | 0 | | 14 | | MM EMERGENCY DEPT | | | 1 | | 0  (acute cholecystitis) | | 234 | | 1 | | 0 | | NA | | NA | | NA | | NA | | 339 | | 0 | | 0 | | 0 | | 0 | | 0 | | 0 | | 0 | | 0 | |
| P5788 | 0 | | 13 | | MM EMERGENCY DEPT | | | 1 | | 2 | | 215 | | 1 | | 0 | | NA | | NA | | NA | | NA | | 367 | | 0 | | 0 | | 0 | | 0 | | 0 | | 0 | | 0 | | 0 | |
| P7208 | 0 | | 13 | | MM EMERGENCY DEPT | | | 1 | | 2 | | 215 | | 1 | | 0 | | NA | | NA | | NA | | NA | | 236 | | 0 | | 0 | | 0 | | 0 | | 0 | | 0 | | 0 | | 0 | |
| P4250 | 1 | | 0 | | MM PICU | | | 1 | | 6 | | 660 | | 1 | | 0 | | 0.9 | | 10.7 | | 26 | | 2.1 | | 504 | | 0 | | 0 | | 0 | | 0 | | 0 | | 0 | | 0 | | 0 | |
| P7209 | 0 | | 13 | | MM EMERGENCY DEPT | | | 1 | | 2 | | 215 | | 1 | | 0 | | 1.2 | | 14 | | NA | | NA | | 219 | | 0 | | 0 | | 0 | | 0 | | 0 | | 0 | | 0 | | 0 | |
| P5418 | 0 | | 13 | | MM EMERGENCY DEPT | | | 1 | | 1 | | 355 | | 1 | | 0 | | 1.2 | | 14 | | 35 | | 5.9 | | 213 | | 0 | | 0 | | 0 | | 1  (doppler US) | | 0 | | 0 | | 0 | | 0 | |
| P3442 | 0 | | 13 | | MM EMERGENCY DEPT | | | 1 | | 2 | | 541 | | 1 | | 0 | | NA | | NA | | NA | | NA | | 307 | | 0 | | 0 | | 0 | | 1  (CTPA) | | 0 | | 0 | | 0 | | 0 | |
| P352 | 0 | | 13 | | MM 3Z2 MEDICINE | | | 1 | | 10 | | 119000 | | 2 | | 8 | | 1.1 | | 13.3 | | 33 | | 7.4 | | 478 | | 0 | | 0 | | 1  (PE) | | 1  (CTPA at SickKids) | | 0 | | 0 | | 1 | | 0 | |
| P867 | 1 | | 11 | | MM EMERGENCY DEPT | | | 1 | | 0  (fatty liver) | | 299 | | 1 | | 0 | | 1.1 | | 12.3 | | NA | | 4 | | 351 | | 0 | | 0 | | 0 | | 0 | | 1 | | 0 | | 0 | | 0 | |
| P5786 | 1 | | 0 | | MM EMERGENCY DEPT | | | 1 | | 6 | | 1000 | | 2 | | 0 | | 1.5 | | 17.6 | | 30 | | 5.6 | | 625 | | 0 | | 0 | | 0 | | 0 | | 0 | | 0 | | 0 | | 0 | |
| P6539 | 1 | | 11 | | MM EMERGENCY DEPT | | | 1 | | 3 | | 302 | | 1 | | 0 | | 1.1 | | 12.1 | | NA | | NA | | 314 | | 0 | | 0 | | 0 | | 1  (head MRI or CT) | | 0 | | 0 | | 0 | | 0 | |
| P339 | 0 | | 14 | | JH EMERGENCY DEPT | | | 1 | | 1 | | 215 | | 1 | | 0 | | NA | | NA | | NA | | NA | | 265 | | 0 | | 0 | | 0 | | 0 | | 0 | | 0 | | 0 | | 0 | |
| P860 | 0 | | 14 | | MM EMERGENCY DEPT | | | 1 | | 2 | | 215 | | 1 | | 0 | | 1 | | 11 | | 30 | | NA | | 286 | | 0 | | 0 | | 0 | | 0 | | 0 | | 0 | | 0 | | 0 | |
| P5411 | 0 | | 14 | | MM EMERGENCY DEPT | | | 1 | | 2 | | 215 | | 1 | | 0 | | NA | | NA | | NA | | NA | | 302 | | 0 | | 0 | | 0 | | 0 | | 0 | | 0 | | 0 | | 0 | |
| P8934 | 0 | | 14 | | MM EMERGENCY DEPT | | | 1 | | 2 | | 234 | | 1 | | 0 | | NA | | NA | | NA | | NA | | 270 | | 0 | | 1  (PERC) | | 0 | | 0 | | 0 | | 0 | | 0 | | 0 | |
| P5783 | 0 | | 14 | | MM EMERGENCY DEPT | | | 1 | | 1 | | 338 | | 1 | | 0 | | NA | | NA | | NA | | 3 | | 309 | | 0 | | 0 | | 0 | | 1  (doppler US) | | 0 | | 0 | | 0 | | 0 | |
| P6319 | 0 | | 14 | | HGH EMERGENCY DEPT | | | 1 | | 2 | | 340 | | 1 | | 0 | | 1.3 | | 14.6 | | NA | | NA | | 288 | | 0 | | 0 | | 0 | | 0 | | 0 | | 0 | | 0 | | 0 | |
| P5779 | 0 | | 14 | | MM EMERGENCY DEPT | | | 1 | | 2 | | 482 | | 1 | | 0 | | 1 | | 10.8 | | NA | | NA | | 267 | | 0 | | 0 | | 0 | | 0 | | 0 | | 0 | | 0 | | 0 | |
| P6134 | 0 | | 14 | | MM EMERGENCY DEPT | | | 1 | | 2 | | 789 | | 2 | | 2 | | 1.1 | | 12.1 | | NA | | NA | | NA | | 0 | | 0 | | 0 | | 1  (doppler US) | | 0 | | 0 | | 0 | | 0 | |
| P6872 | 1 | | 14 | | MM EMERGENCY DEPT | | | 1 | | 2 | | 219 | | 1 | | 0 | | NA | | NA | | NA | | NA | | 507 | | 0 | | 0 | | 0 | | 0 | | 0 | | 0 | | 0 | | 0 | |
| P4260 | 1 | | 14 | | MM 3Z2 MEDICINE | | | 0 | | 2 | | 4030 | | 2 | | 0 | | 1.3 | | 15.5 | | 23 | | 1.3 | | 277 | | 0 | | 0 | | 0 | | 0 | | 0 | | 0 | | 0 | | 0 | |
| P1280 | 0 | | 0 | | MM NICU | | | 1 | | 4 | | 379 | | 1 | | 0 | | 4.6 | | 54 | | >200 | | <0.4 | | 107 | | 0 | | 0 | | 0 | | 0 | | 1 | | 1 | | 0 | | 0 | |
| P4675 | 1 | | 14 | | MM EMERGENCY DEPT | | | 1 | | 2 | | 624 | | 2 | | 0 | | 1.1 | | 12.6 | | 30 | | NA | | 142 | | 0 | | 0 | | 0 | | 0 | | 0 | | 0 | | 0 | | 0 | |
| P3433 | 1 | | 14 | | MM LAB SCC | | | 1 | | 1 | | 598 | | 2 | | 0 | | 1.7 | | 19.7 | | NA | | NA | | 117 | | 0 | | 0 | | 0 | | 1  (doppler US) | | 0 | | 0 | | 0 | | 0 | |
| P5062 | 1 | | 0 | | MM PICU | | | 1 | | 6 | | 1090 | | 2 | | 0 | | 0.8 | | 9.4 | | 32 | | 2.7 | | 335 | | 0 | | 0 | | 0 | | 0 | | 0 | | 0 | | 1 | | 0 | |
| P5781 | 1 | | 3 | | MM EMERGENCY DEPT | | | 1 | | 4 | | 401 | | 1 | | 0 | | 1.3 | | 14.2 | | 33 | | 2.6 | | 310 | | 0 | | 0 | | 1  (stroke) | | 1  (head MRI or CT) | | 0 | | 0 | | 0 | | 0 | |
| P6100 | 0 | | 15 | | JH EMERGENCY DEPT | | | 1 | | 2 | | 215 | | 1 | | 0 | | NA | | NA | | NA | | NA | | 316 | | 0 | | 0 | | 0 | | 0 | | 0 | | 0 | | 0 | | 0 | |
| P4667 | 1 | | 15 | | MM IR DEP | | | 1 | | 4  (localized) | | 416 | | 1 | | 0 | | NA | | NA | | NA | | 2.6 | | NA | | 0 | | 0 | | 0 | | 0 | | 0 | | 0 | | 0 | | 0 | |
| P1716 | 0 | | 15 | | MM EMERGENCY DEPT | | | 1 | | 2 | | 242 | | 1 | | 0 | | NA | | NA | | NA | | NA | | 378 | | 0 | | 0 | | 0 | | 0 | | 0 | | 0 | | 0 | | 0 | |
| P6125 | 0 | | 15 | | JH EMERGENCY DEPT | | | 1 | | 2 | | 279 | | 1 | | 0 | | NA | | NA | | NA | | NA | | 373 | | 0 | | 0 | | 0 | | 0 | | 0 | | 0 | | 0 | | 0 | |
| P6143 | 1 | | 15 | | MM PED HEMATOLOGY | | | 1 | | 0  (hemophilia B clinic) | | 422 | | 1 | | 0 | | 1.2 | | 13.3 | | 42 | | 3.6 | | 254 | | 0 | | 0 | | 0 | | 0 | | 0 | | 0 | | 0 | | 0 | |
| P6537 | 1 | | 0 | | MM PICU | | | 1 | | 4 | | 438 | | 1 | | 0 | | 1.2 | | 13 | | 42 | | NA | | 208 | | 0 | | 0 | | 0 | | 0 | | 0 | | 0 | | 0 | | 0 | |
| P5051 | 1 | | 5 | | MM EMERGENCY DEPT | | | 1 | | 0  (Vit K deficiency/ low FVII) | | 452 | | 1 | | 0 | | 1.7 | | 19.4 | | 37 | | 3 | | 188 | | 0 | | 0 | | 0 | | 0 | | 0 | | 0 | | 0 | | 0 | |
| P1301 | 0 | | 15 | | MM EMERGENCY DEPT | | | 1 | | 2 | | 286 | | 1 | | 0 | | NA | | NA | | NA | | NA | | 266 | | 0 | | 0 | | 0 | | 0 | | 0 | | 0 | | 0 | | 0 | |
| P5064 | 0 | | 15 | | MM EMERGENCY DEPT | | | 1 | | 2 | | 307 | | 1 | | 0 | | 1.1 | | 13 | | NA | | NA | | 389 | | 0 | | 0 | | 0 | | 0 | | 0 | | 0 | | 0 | | 0 | |
| P2579 | 0 | | 15 | | MM EMERGENCY DEPT | | | 1 | | 2 | | 410 | | 1 | | 0 | | 1 | | 12 | | NA | | NA | | 233 | | 0 | | 0 | | 0 | | 0 | | 0 | | 0 | | 0 | | 0 | |
| P6533 | 1 | | 0 | | MM PICU | | | 1 | | 6 | | 1360 | | 2 | | 1 | | 1.8 | | 20.1 | | 39 | | 4.7 | | 677 | | 0 | | 0 | | 0 | | 0 | | 0 | | 0 | | 0 | | 0 | |
| P6538 | 0 | | 15 | | MM EMERGENCY DEPT | | | 1 | | 2 | | 419 | | 1 | | 0 | | 1.1 | | 13.2 | | NA | | NA | | 413 | | 0 | | 0 | | 0 | | 1  (CTPA) | | 0 | | 0 | | 0 | | 0 | |
| P1711 | 0 | | 15 | | MM EMERGENCY DEPT | | | 1 | | 2 | | 514 | | 1 | | 0 | | NA | | NA | | NA | | NA | | 151 | | 0 | | 0 | | 0 | | 0 | | 0 | | 0 | | 0 | | 0 | |
| P5048 | 0 | | 15 | | MM 3B2 HEM/ONC | | | 1 | | 2 | | 2920 | | 2 | | 0 | | 0.9 | | 10.6 | | NA | | 1.4 | | 43 | | 0 | | 0 | | 0 | | 1  (CTPA) | | 1 | | 1 | | 0 | | 1  (ALL) | |
| P6893 | 0 | | 14 | | MM EMERGENCY DEPT | | | 1 | | 4 | | 532 | | 1 | | 0 | | 1.3 | | 14.6 | | 29 | | 4.7 | | 7 | | 0 | | 0 | | 0 | | 0 | | 0 | | 0 | | 0 | | 0 | |
| P2593 | 0 | | 15 | | MM EMERGENCY DEPT | | | 1 | | 1 | | 711 | | 2 | | 0 | | NA | | NA | | NA | | NA | | 338 | | 0 | | 0 | | 0 | | 1  (doppler US) | | 0 | | 0 | | 1 | | 0 | |
| P5425 | 0 | | 15 | | MM EMERGENCY DEPT | | | 1 | | 10 | | 14100 | | 2 | | 3 | | 1.1 | | 12.6 | | 26 | | 5.6 | | 205 | | 0 | | 0 | | 1  (DVT/PE) | | 1  (CTPA/  doppler) | | 0 | | 0 | | 0 | | 0 | |
| P3420 | 1 | | 15 | | MM EMERGENCY DEPT | | | 1 | | 2 | | 215 | | 1 | | 0 | | 1 | | 11.8 | | NA | | NA | | 117 | | 0 | | 0 | | 0 | | 0 | | 0 | | 0 | | 0 | | 0 | |
| P5777 | 1 | | 4 | | MM 3C MEDICINE | | | 1 | | 4 | | 606 | | 1 | | 3 | | 0.9 | | 10.5 | | 19 | | 1.9 | | 451 | | 0 | | 0 | | 0 | | 0 | | 0 | | 0 | | 0 | | 0 | |
| P5044 | 1 | | 15 | | MM 3Y1 SURGERY | | | 1 | | 10 | | 759 | | 2 | | 0 | | 1.4 | | 15.8 | | 34 | | NA | | 474 | | 0 | | 0 | | 1  (CSVT) | | 1  (head MRI or CT) | | 0 | | 0 | | 1 | | 0 | |
| P861 | 0 | | 16 | | MM EMERGENCY DEPT | | | 1 | | 2 | | 215 | | 1 | | 0 | | NA | | NA | | NA | | NA | | 253 | | 0 | | 0 | | 0 | | 0 | | 0 | | 0 | | 0 | | 0 | |
| P1895 | 0 | | 16 | | HGH EMERGENCY DEPT | | | 1 | | 2 | | 215 | | 1 | | 0 | | NA | | NA | | NA | | NA | | 520 | | 0 | | 1  (PERC) | | 0 | | 0 | | 0 | | 0 | | 0 | | 0 | |
| P7211 | 1 | | 0 | | MM PICU | | | 1 | | 6 | | 4360 | | 2 | | 1 | | 1 | | 11.3 | | 33 | | 2.2 | | 47 | | 0 | | 0 | | 0 | | 0 | | 1 | | 0 | | 0 | | 0 | |
| P1300 | 1 | | 1 | | MM PICU | | | 1 | | 3 | | 644 | | 1 | | 0 | | 1.2 | | 13.6 | | 33 | | 2.9 | | 372 | | 0 | | 0 | | 1  (stroke) | | 1  (head MRI or CT) | | 0 | | 0 | | 0 | | 0 | |
| P6386 | 0 | | 16 | | HGH EMERGENCY DEPT | | | 1 | | 2 | | 219 | | 1 | | 0 | | 1.1 | | 11.8 | | NA | | NA | | 316 | | 0 | | 1  (Wells & PERC) | | 0 | | 0 | | 0 | | 0 | | 0 | | 0 | |
| P9041 | 1 | | 0 | | MM 3C MEDICINE | | | 1 | | 6 | | 6910 | | 2 | | 0 | | 1 | | 11.6 | | NA | | 5 | | 133 | | 0 | | 0 | | 0 | | 0 | | 0 | | 0 | | 0 | | 0 | |
| P5059 | 0 | | 0 | | MM PICU | | | 1 | | 4 | | 670 | | 1 | | 0 | | 1.4 | | 16.5 | | 37 | | 3.9 | | 480 | | 0 | | 0 | | 0 | | 0 | | 0 | | 0 | | 0 | | 0 | |
| P9042 | 1 | | 2 | | MM EMERGENCY DEPT | | | 1 | | 0  (bleeding investigation/ no clear diagnosis) | | 681 | | 1 | | 0 | | 1.1 | | 13.1 | | 35 | | 2 | | 283 | | 0 | | 0 | | 0 | | 0 | | 0 | | 0 | | 0 | | 0 | |
| P1302 | 0 | | 1 | | MM EMERGENCY DEPT | | | 1 | | 6 | | 482 | | 1 | | 0 | | 1 | | 12.1 | | 24 | | 4 | | NA | | 0 | | 0 | | 0 | | 0 | | 0 | | 0 | | 0 | | 0 | |
| P4342 | 0 | | 16 | | SJHK URGENT CARE | | | 1 | | 2 | | 264 | | 1 | | 0 | | NA | | NA | | NA | | NA | | 436 | | 0 | | 0 | | 0 | | 0 | | 0 | | 0 | | 0 | | 0 | |
| P6535 | 1 | | 14 | | MM EMERGENCY DEPT | | | 1 | | 3 | | 736 | | 2 | | 0 | | 1.1 | | 12.3 | | 24 | | 2.6 | | 222 | | 0 | | 0 | | 1  (stroke) | | 1  (head MRI or CT) | | 0 | | 0 | | 0 | | 0 | |
| P5080 | 0 | | 16 | | SJHK URGENT CARE | | | 1 | | 2 | | 319 | | 1 | | 0 | | NA | | NA | | NA | | NA | | 395 | | 0 | | 1  (Wells & PERC) | | 0 | | 0 | | 0 | | 0 | | 0 | | 0 | |
| P3417 | 1 | | 3 | | MM EMERGENCY DEPT | | | 1 | | 4 | | 765 | | 1 | | 0 | | 1.1 | | 13 | | 40 | | 3.6 | | 1 | | 0 | | 0 | | 0 | | 0 | | 0 | | 0 | | 0 | | 0 | |
| P5426 | 0 | | 16 | | MM EMERGENCY DEPT | | | 1 | | 2 | | 362 | | 1 | | 0 | | NA | | NA | | NA | | NA | | 238 | | 0 | | 0 | | 0 | | 0 | | 0 | | 0 | | 0 | | 0 | |
| P844 | 0 | | 2 | | MM EMERGENCY DEPT | | | 1 | | 4 | | 797 | | 2 | | 0 | | 1.7 | | 19.4 | | 30 | | 2.1 | | 11 | | 0 | | 0 | | 0 | | 0 | | 0 | | 0 | | 1 | | 0 | |
| P3010 | 0 | | 16 | | MM EMERGENCY DEPT | | | 1 | | 2 | | 2010 | | 2 | | 0 | | NA | | NA | | NA | | NA | | 262 | | 0 | | 0 | | 0 | | 0 | | 0 | | 0 | | 0 | | 0 | |
| P4684 | 1 | | 1 | | MM EMERGENCY DEPT | | | 1 | | 6 | | 629 | | 1 | | 0 | | 1.3 | | 15.3 | | NA | | 2.7 | | 371 | | 0 | | 0 | | 0 | | 0 | | 0 | | 0 | | 0 | | 0 | |
| P864 | 0 | | 16 | | MM 3C MEDICINE | | | 1 | | 2 | | 1370 | | 2 | | 0 | | 1 | | 12 | | 41 | | NA | | 305 | | 0 | | 0 | | 0 | | 0 | | 0 | | 1 | | 0 | | 0 | |
| P1715 | 0 | | 16 | | MM EMERGENCY DEPT | | | 1 | | 2 | | 522 | | 2 | | 0 | | NA | | NA | | NA | | NA | | 240 | | 0 | | 1  (Wells) | | 0 | | 1  (CTPA) | | 0 | | 0 | | 0 | | 0 | |
| P850 | 1 | | 1 | | MM PICU | | | 1 | | 6 | | 979 | | 2 | | 1 | | 0.9 | | 10.9 | | 22 | | 2.4 | | 355 | | 0 | | 0 | | 0 | | 0 | | 0 | | 0 | | 0 | | 0 | |
| P3852 | 0 | | 1 | | MM EMERGENCY DEPT | | | 1 | | 7  (KD/MIS-C) | | 1140 | | 2 | | 0 | | NA | | NA | | NA | | NA | | 298 | | 0 | | 0 | | 0 | | 0 | | 0 | | 0 | | 0 | | 0 | |
| P5328 | 1 | | 16 | | JH EMERGENCY DEPT | | | 1 | | 1 | | 215 | | 1 | | 0 | | NA | | NA | | NA | | NA | | 203 | | 0 | | 0 | | 0 | | 0 | | 0 | | 0 | | 1 | | 0 | |
| P3856 | 1 | | 1 | | MM EMERGENCY DEPT | | | 1 | | 7  (KD/MIS-C) | | 1990 | | 2 | | 2 | | NA | | NA | | NA | | NA | | 133 | | 0 | | 0 | | 0 | | 0 | | 0 | | 0 | | 0 | | 0 | |
| P1305 | 1 | | 1 | | MM EMERGENCY DEPT | | | 1 | | 6 | | 16200 | | 2 | | 2 | | 1.1 | | 13 | | 37 | | 3.5 | | 221 | | 0 | | 0 | | 0 | | 0 | | 0 | | 1 | | 0 | | 0 | |
| P4678 | 1 | | 3 | | MM EMERGENCY DEPT | | | 1 | | 7  (KD/MIS-C) | | 820 | | 2 | | 5 | | 1.6 | | 18.9 | | 37 | | 7.6 | | 224 | | 0 | | 0 | | 0 | | 0 | | 0 | | 0 | | 0 | | 0 | |
| P3570 | 1 | | 16 | | SJHC 1 EMERGENCY | | | 1 | | 2 | | 215 | | 1 | | 0 | | NA | | NA | | NA | | NA | | 282 | | 0 | | 0 | | 0 | | 0 | | 0 | | 0 | | 0 | | 0 | |
| P3874 | 1 | | 0 | | MM EMERGENCY DEPT | | | 1 | | 0  (lymphoid neoplasm) | | 972 | | 2 | | 0 | | 1.1 | | 12.5 | | 33 | | NA | | 79 | | 0 | | 0 | | 0 | | 0 | | 0 | | 0 | | 0 | | 1  (lymphoid neoplasm) | |
| P858 | 0 | | 3 | | MM EMERGENCY DEPT | | | 1 | | 7  (KD/MIS-C) | | 919 | | 2 | | 0 | | 1.3 | | 14.7 | | 33 | | NA | | 208 | | 0 | | 0 | | 0 | | 0 | | 1 | | 0 | | 0 | | 0 | |
| P6882 | 1 | | 0 | | MM 3Y1 SURGERY | | | 1 | | 0  (bleeding investigation/ no diagnosis) | | 996 | | 2 | | 0 | | 1.2 | | 13.9 | | 36 | | 1.8 | | 801 | | 0 | | 0 | | 0 | | 0 | | 0 | | 0 | | 0 | | 0 | |
| P3876 | 1 | | 3 | | MM 3C MEDICINE | | | 1 | | 7  (KD/MIS-C) | | 1260 | | 2 | | 0 | | 1.3 | | 14.6 | | 30 | | NA | | 468 | | 0 | | 0 | | 0 | | 0 | | 0 | | 0 | | 0 | | 0 | |
| P1719 | 0 | | 3 | | MM PICU | | | 1 | | 7  (KD/MIS-C) | | 4790 | | 2 | | 4 | | 1.1 | | 12.8 | | 34 | | 4 | | 92 | | 0 | | 0 | | 0 | | 0 | | 0 | | 0 | | 0 | | 0 | |
| P7222 | 1 | | 4 | | MM EMERGENCY DEPT | | | 1 | | 7  (KD/MIS-C) | | 926 | | 2 | | 0 | | 1.2 | | 13.6 | | 34 | | NA | | 217 | | 0 | | 0 | | 0 | | 0 | | 1 | | 0 | | 0 | | 0 | |
| P5778 | 1 | | 16 | | MM EMERGENCY DEPT | | | 1 | | 2 | | 215 | | 1 | | 0 | | 1.2 | | 13.5 | | NA | | NA | | 259 | | 0 | | 0 | | 0 | | 0 | | 0 | | 0 | | 0 | | 0 | |
| P7206 | 0 | | 5 | | MM 3Z2 MEDICINE | | | 1 | | 4 | | 1100 | | 2 | | 0 | | 1.7 | | 19 | | 39 | | 2.7 | | 96 | | 0 | | 0 | | 0 | | 0 | | 0 | | 0 | | 0 | | 0 | |
| P6140 | 1 | | 16 | | MM EMERGENCY DEPT | | | 1 | | 2 | | 215 | | 1 | | 0 | | NA | | NA | | NA | | NA | | 206 | | 0 | | 0 | | 0 | | 0 | | 0 | | 0 | | 0 | | 0 | |
| P6548 | 0 | | 0 | | MM NICU | | | 1 | | 0  (Vit K deficiency/ low FVII) | | 1140 | | 1 | | 0 | | 3.3 | | 39.2 | | 42 | | 2 | | 233 | | 0 | | 0 | | 0 | | 0 | | 0 | | 0 | | 0 | | 0 | |
| P3875 | 0 | | 4 | | MM EMERGENCY DEPT | | | 1 | | 7  (KD/MIS-C) | | 1680 | | 2 | | 0 | | NA | | NA | | NA | | 7.2 | | 345 | | 0 | | 0 | | 0 | | 0 | | 0 | | 0 | | 0 | | 0 | |
| P6887 | 0 | | 7 | | MM LAB SCC | | | 1 | | 4  (localized) | | 1210 | | 2 | | 0 | | 1.1 | | 12.6 | | 36 | | 2.3 | | 303 | | 0 | | 0 | | 0 | | 1  (doppler US) | | 0 | | 0 | | 0 | | 0 | |
| P3863 | 1 | | 16 | | MM IR DEP | | | 1 | | 1 | | 228 | | 1 | | 0 | | 1.1 | | 12.2 | | NA | | 3 | | 187 | | 0 | | 0 | | 0 | | 0 | | 0 | | 0 | | 0 | | 0 | |
| P4256 | 1 | | 16 | | MM EMERGENCY DEPT | | | 1 | | 8 | | 391 | | 1 | | 0 | | NA | | NA | | NA | | NA | | 256 | | 0 | | 0 | | 0 | | 0 | | 0 | | 0 | | 0 | | 0 | |
| P4757 | 1 | | 16 | | SJHK URGENT CARE | | | 1 | | 2 | | 1240 | | 2 | | 0 | | NA | | NA | | NA | | NA | | 172 | | 0 | | 0 | | 0 | | 1  (CTPA) | | 0 | | 0 | | 0 | | 0 | |
| P3853 | 0 | | 4 | | MM LAB SCC | | | 1 | | 7  (sJIA/active) | | 24000 | | 2 | | 0 | | 1.3 | | 15.5 | | 39 | | 4.9 | | 739 | | 0 | | 0 | | 0 | | 0 | | 0 | | 0 | | 0 | | 0 | |
| P1292 | 0 | | 6 | | MM PICU | | | 1 | | 6 | | 889 | | 2 | | 4 | | 1.7 | | 19.2 | | 37 | | 2 | | 216 | | 0 | | 0 | | 0 | | 0 | | 0 | | 0 | | 0 | | 0 | |
| P357 | 1 | | 0 | | MM NICU | | | 1 | | 3 | | 1290 | | 2 | | 0 | | 1.1 | | 13.3 | | 43 | | 2 | | 122 | | 0 | | 0 | | 1  (stroke) | | 1  (head MRI or CT) | | 0 | | 0 | | 0 | | 0 | |
| P351 | 0 | | 6 | | MM EMERGENCY DEPT | | | 1 | | 7  (KD/MIS-C) | | 1560 | | 2 | | 1 | | 1.1 | | 12.5 | | 24 | | 1.6 | | 45 | | 0 | | 0 | | 0 | | 0 | | 0 | | 0 | | 0 | | 0 | |
| P5680 | 1 | | 16 | | MM EMERGENCY DEPT | | | 1 | | 1 | | 907 | | 2 | | 0 | | NA | | NA | | NA | | NA | | 205 | | 0 | | 0 | | 0 | | 1  (doppler US) | | 0 | | 0 | | 0 | | 0 | |
| P1721 | 0 | | 0 | | MM NICU | | | 1 | | 4 | | 1340 | | 2 | | 0 | | 1.5 | | 17.3 | | 34 | | 1.4 | | 32 | | 0 | | 0 | | 0 | | 0 | | 0 | | 1 | | 0 | | 0 | |
| P1714 | 0 | | 6 | | MM 3C MEDICINE | | | 1 | | 7  (KD/MIS-C) | | 5520 | | 2 | | 1 | | NA | | NA | | NA | | NA | | 225 | | 0 | | 0 | | 0 | | 0 | | 0 | | 0 | | 0 | | 0 | |
| P1286 | 1 | | 16 | | MM EMERGENCY DEPT | | | 1 | | 2 | | 881 | | 2 | | 0 | | 1.7 | | 19.9 | | 34 | | NA | | 130 | | 0 | | 0 | | 0 | | 1  (CTPA) | | 0 | | 0 | | 0 | | 0 | |
| P5043 | 1 | | 3 | | MM PICU | | | 0 | | 4 | | 1490 | | 2 | | 0 | | 1.8 | | 20.5 | | 24 | | 5.9 | | 2 | | 0 | | 0 | | 0 | | 0 | | 0 | | 0 | | 0 | | 0 | |
| P5421 | 1 | | 7 | | MM PICU | | | 1 | | 7  (KD/MIS-C) | | 2350 | | 2 | | 1 | | 1.3 | | 14.7 | | 37 | | 8.8 | | 238 | | 0 | | 0 | | 0 | | 0 | | 0 | | 0 | | 0 | | 0 | |
| P5780 | 1 | | 0 | | MM NICU | | | 1 | | 3 | | 1630 | | 2 | | 0 | | 1.4 | | 16.5 | | 54 | | 1.6 | | 45 | | 0 | | 0 | | 0 | | 1  (arterial doppler) | | 0 | | 0 | | 0 | | 0 | |
| P6545 | 1 | | 4 | | MM PICU | | | 1 | | 3 | | 1630 | | 2 | | 20 | | 1.3 | | 14.4 | | 30 | | 2.4 | | 117 | | 0 | | 0 | | 1  (femoral artery) | | 1  (arterial doppler) | | 1 | | 1 | | 1 | | 0 | |
| P360 | 0 | | 0 | | MM PICU | | | 1 | | 4 | | 1640 | | 2 | | 0 | | 1.5 | | 17.6 | | 30 | | 0.4 | | 14 | | 0 | | 0 | | 0 | | 0 | | 0 | | 0 | | 0 | | 0 | |
| P6534 | 1 | | 7 | | MM EMERGENCY DEPT | | | 1 | | 7  (sJIA) | | 3590 | | 2 | | 0 | | 1.1 | | 12.3 | | NA | | NA | | 273 | | 0 | | 0 | | 0 | | 1  (knee X-ray) | | 0 | | 0 | | 0 | | 0 | |
| P2575 | 1 | | 0 | | MM NICU | | | 1 | | 4 | | 1750 | | 2 | | 0 | | 1.6 | | 18.6 | | 60 | | NA | | 212 | | 0 | | 0 | | 0 | | 0 | | 0 | | 0 | | 0 | | 0 | |
| P4251 | 1 | | 12 | | MM MAIN OR | | | 1 | | 4 | | 1770 | | 2 | | 0 | | 1.5 | | 20.1 | | 38 | | 4.7 | | 104 | | 0 | | 0 | | 0 | | 1  (head MRI or CT) | | 0 | | 0 | | 1 | | 0 | |
| P3423 | 1 | | 8 | | MM EMERGENCY DEPT | | | 1 | | 6 | | 256 | | 1 | | 4 | | 1.1 | | 13.2 | | 30 | | 2.1 | | 46 | | 0 | | 0 | | 0 | | 0 | | 0 | | 0 | | 0 | | 0 | |
| P4676 | 0 | | 0 | | MM NICU | | | 1 | | 4  (localized) | | 1950 | | 2 | | 0 | | 1 | | 11.6 | | 41 | | 3 | | 143 | | 0 | | 0 | | 0 | | 0 | | 0 | | 0 | | 0 | | 0 | |
| P3859 | 0 | | 8 | | MM EMERGENCY DEPT | | | 1 | | 7  (KD/MIS-C) | | 6540 | | 2 | | 0 | | 1.2 | | 13.9 | | 31 | | 4 | | 194 | | 0 | | 0 | | 0 | | 0 | | 0 | | 0 | | 0 | | 0 | |
| P2585 | 1 | | 9 | | MM EMERGENCY DEPT | | | 1 | | 7  (KD/MIS-C) | | 1960 | | 2 | | 0 | | 1.1 | | 13.1 | | 27 | | 9.5 | | 337 | | 0 | | 0 | | 0 | | 0 | | 1 | | 0 | | 0 | | 0 | |
| P4490 | 0 | | 17 | | HGH EMERGENCY DEPT | | | 1 | | 1 | | 215 | | 1 | | 0 | | NA | | NA | | NA | | NA | | 270 | | 0 | | 0 | | 0 | | 0 | | 0 | | 0 | | 0 | | 0 | |
| P5412 | 1 | | 0 | | MM NICU | | | 1 | | 3 | | 2020 | | 2 | | 9 | | 1.6 | | 18.7 | | 121 | | 2.7 | | 155 | | 0 | | 0 | | 1  (brachial artery) | | 1  (arterial doppler) | | 0 | | 0 | | 0 | | 0 | |
| P6152 | 0 | | 9 | | MM EMERGENCY DEPT | | | 1 | | 7  (KD/MIS-C) | | 4140 | | 2 | | 2 | | 1.4 | | 15.4 | | 32 | | 6.6 | | 343 | | 0 | | 0 | | 0 | | 0 | | 0 | | 0 | | 0 | | 0 | |
| P4255 | 0 | | 0 | | MM NICU | | | 1 | | 4 | | 2100 | | 2 | | 0 | | 1.6 | | 19 | | >200 | | 2.2 | | 97 | | 0 | | 0 | | 1  (portal) | | 1  (abdominal US) | | 0 | | 0 | | 0 | | 0 | |
| P4685 | 1 | | 10 | | MM 3B2 HEM/ONC | | | 1 | | 7  (KD/MIS-C) | | 887 | | 2 | | 1 | | 1.1 | | 13.2 | | 34 | | 1.8 | | 134 | | 0 | | 0 | | 0 | | 0 | | 1 | | 0 | | 0 | | 1  (B-ALL post SCT) | |
| P356 | 0 | | 17 | | MM EMERGENCY DEPT | | | 1 | | 2 | | 215 | | 1 | | 0 | | 1.2 | | 13.5 | | NA | | NA | | 210 | | 0 | | 0 | | 0 | | 0 | | 0 | | 0 | | 0 | | 0 | |
| P4257 | 0 | | 3 | | MM 3Z2 MEDICINE | | | 1 | | 4 | | 3060 | | 2 | | 2 | | 1.2 | | 14.3 | | 20 | | 3.4 | | 401 | | 0 | | 0 | | 0 | | 0 | | 0 | | 0 | | 0 | | 0 | |
| P3854 | 0 | | 17 | | MM EMERGENCY DEPT | | | 1 | | 2 | | 215 | | 1 | | 0 | | NA | | NA | | NA | | NA | | 175 | | 0 | | 0 | | 0 | | 0 | | 0 | | 0 | | 0 | | 0 | |
| P5049 | 0 | | 10 | | MM EMERGENCY DEPT | | | 1 | | 7  (KD/MIS-C) | | 1870 | | 2 | | 1 | | 2 | | 23.5 | | 32 | | 5.6 | | 247 | | 0 | | 0 | | 0 | | 0 | | 0 | | 0 | | 0 | | 0 | |
| P2734 | 0 | | 0 | | SJHC 3 SPEC CARE NURS | | | 1 | | 4 | | 4000 | | 2 | | 0 | | 1.1 | | 13.1 | | 42 | | 1.8 | | 18 | | 0 | | 0 | | 0 | | 0 | | 0 | | 0 | | 0 | | 0 | |
| P6881 | 0 | | 17 | | MM EMERGENCY DEPT | | | 1 | | 2 | | 215 | | 1 | | 0 | | NA | | NA | | NA | | NA | | NA | | 0 | | 0 | | 0 | | 1  (doppler US) | | 0 | | 0 | | 0 | | 0 | |
| P3851 | 0 | | 10 | | MM EMERGENCY DEPT | | | 1 | | 7  (KD/MIS-C) | | 2050 | | 2 | | 1 | | 1.4 | | 15.8 | | 35 | | 2.6 | | 63 | | 0 | | 0 | | 0 | | 0 | | 0 | | 0 | | 0 | | 0 | |
| P2589 | 1 | | 11 | | MM PICU | | | 1 | | 6 | | 914 | | 2 | | 1 | | 1.1 | | 12.5 | | 31 | | 6.8 | | 121 | | 0 | | 0 | | 0 | | 0 | | 0 | | 0 | | 0 | | 0 | |
| P6151 | 0 | | 17 | | MM LAB SCC | | | 1 | | 10 | | 246 | | 1 | | 0 | | 1.8 | | 10.5 | | 45 | | 2.3 | | 271 | | 0 | | 0 | | 0 | | 1  (doppler US) | | 0 | | 0 | | 0 | | 0 | |
| P1282 | 0 | | 12 | | MM PED ONC TMNT | | | 1 | | 5  (post CART) | | 395 | | 1 | | 0 | | 1 | | 12.1 | | 32 | | 3.8 | | 228 | | 0 | | 0 | | 0 | | 0 | | 0 | | 0 | | 0 | | 1  (B-ALL post CART) | |
| P1706 | 1 | | 0 | | MM EMERGENCY DEPT | | | 1 | | 4 | | 5190 | | 2 | | 0 | | 1.1 | | 12.4 | | 37 | | 2.3 | | 138 | | 0 | | 0 | | 0 | | 0 | | 0 | | 1 | | 0 | | 0 | |
| P361 | 1 | | 13 | | MM EMERGENCY DEPT | | | 1 | | 7  (KD/MIS-C) | | 1050 | | 2 | | 1 | | 1.3 | | 15 | | 33 | | 3.3 | | 192 | | 0 | | 0 | | 0 | | 0 | | 1 | | 0 | | 0 | | 0 | |
| P2182 | 0 | | 15 | | MM PICU | | | 1 | | 6 | | 215 | | 1 | | 0 | | 1 | | 11.1 | | 33 | | 3 | | 225 | | 0 | | 0 | | 0 | | 0 | | 0 | | 0 | | 0 | | 0 | |
| P7111 | 0 | | 2 | | MM 3Z2 MEDICINE | | | 1 | | 4 | | 5720 | | 2 | | 2 | | 1.3 | | 14.3 | | 35 | | 3.1 | | 61 | | 0 | | 0 | | 1  (IJV) | | 1  (doppler US) | | 0 | | 1 | | 0 | | 0 | |
| P5047 | 1 | | 0 | | MM NICU | | | 1 | | 4 | | 5780 | | 2 | | 3 | | 1.3 | | 15 | | NA | | NA | | 97 | | 0 | | 0 | | 0 | | 0 | | 0 | | 0 | | 0 | | 0 | |
| P6878 | 0 | | 17 | | MM EMERGENCY DEPT | | | 1 | | 2 | | 418 | | 1 | | 0 | | NA | | NA | | NA | | NA | | NA | | 0 | | 0 | | 0 | | 0 | | 0 | | 0 | | 0 | | 0 | |
| P3004 | 1 | | 15 | | MM EMERGENCY DEPT | | | 1 | | 7  (KD/MIS-C) | | 1270 | | 2 | | 1 | | 1.2 | | 13.6 | | 34 | | 7 | | 114 | | 0 | | 0 | | 0 | | 0 | | 0 | | 0 | | 0 | | 0 | |
| P355 | 1 | | 15 | | MM 3Y1 SURGERY | | | 1 | | 6 | | 1290 | | 2 | | 0 | | 0.9 | | 10.5 | | 30 | | NA | | 243 | | 0 | | 0 | | 0 | | 0 | | 0 | | 0 | | 1 | | 0 | |
| P7207 | 1 | | 0 | | MM 3Y1 SURGERY | | | 1 | | 4 | | 7380 | | 2 | | 0 | | 1.6 | | 17.7 | | 67 | | 2 | | 16 | | 0 | | 0 | | 0 | | 0 | | 0 | | 0 | | 0 | | 0 | |
| P6895 | 0 | | 17 | | MM 3C MEDICINE | | | 1 | | 8 | | 4550 | | 2 | | 0 | | 0.9 | | 10.6 | | 32 | | 2.6 | | 219 | | 0 | | 0 | | 0 | | 1  (abdominal US) | | 0 | | 1 | | 0 | | 0 | |
| P5417 | 0 | | 17 | | MM EMERGENCY DEPT | | | 1 | | 2 | | 555 | | 2 | | 0 | | NA | | NA | | NA | | NA | | 237 | | 0 | | 0 | | 0 | | 1  (CTPA) | | 0 | | 0 | | 0 | | 0 | |
| P4252 | 0 | | 7 | | MM PICU | | | 1 | | 4 | | 12500 | | 2 | | 10 | | 1.3 | | 14.6 | | 37 | | 4.2 | | 11 | | 0 | | 0 | | 1  (cephalic) | | 1  (doppler US) | | 1 | | 0 | | 0 | | 0 | |
| P1686 | 1 | | 17 | | JH EMERGENCY DEPT | | | 1 | | 2 | | 215 | | 1 | | 0 | | NA | | NA | | NA | | NA | | 328 | | 0 | | 0 | | 0 | | 0 | | 0 | | 0 | | 0 | | 0 | |
| P2167 | 0 | | 15 | | MM 3C MEDICINE | | | 1 | | 7  (sJIA) | | 5260 | | 2 | | 4 | | 1.4 | | 15.7 | | 31 | | 3.6 | | 113 | | 0 | | 0 | | 0 | | 0 | | 0 | | 0 | | 0 | | 0 | |
| P6897 | 1 | | 1 | | MM PICU | | | 0 | | 4 | | 16800 | | 2 | | 1 | | 2 | | 23 | | 35 | | 3.1 | | 30 | | 0 | | 0 | | 0 | | 0 | | 0 | | 1 | | 0 | | 0 | |
| P5416 | 1 | | 17 | | MM EMERGENCY DEPT | | | 1 | | 2 | | 215 | | 1 | | 0 | | NA | | NA | | NA | | NA | | 243 | | 0 | | 0 | | 0 | | 0 | | 0 | | 0 | | 0 | | 0 | |
| P1707 | 1 | | 14 | | MM 3C MEDICINE | | | 1 | | 4 | | 18200 | | 2 | | 1 | | 1 | | 11.8 | | 27 | | 2.6 | | 52 | | 0 | | 0 | | 0 | | 0 | | 0 | | 0 | | 0 | | 0 | |
| P365 | 1 | | 16 | | MM EMERGENCY DEPT | | | 1 | | 7  (KD/MIS-C) | | 694 | | 2 | | 0 | | 1.2 | | 13.4 | | 31 | | 3.2 | | 175 | | 0 | | 0 | | 0 | | 0 | | 0 | | 0 | | 0 | | 0 | |
| P7219 | 1 | | 1 | | MM PICU | | | 1 | | 4 | | 28700 | | 2 | | 0 | | 1.4 | | 15.5 | | 38 | | 3.1 | | 20 | | 0 | | 0 | | 0 | | 0 | | 0 | | 1 | | 0 | | 0 | |
| P9145 | 1 | | 17 | | MM EMERGENCY DEPT | | | 1 | | 2 | | 365 | | 1 | | 0 | | NA | | NA | | NA | | NA | | 207 | | 0 | | 0 | | 0 | | 0 | | 0 | | 0 | | 0 | | 0 | |
| P4258 | 1 | | 0 | | MM PICU | | | 1 | | 4 | | 32700 | | 2 | | 1 | | 1.5 | | 17.3 | | 32 | | 2.4 | | 37 | | 0 | | 0 | | 0 | | 0 | | 0 | | 0 | | 0 | | 0 | |
| P2584 | 1 | | 17 | | MM EMERGENCY DEPT | | | 1 | | 2 | | 464 | | 1 | | 0 | | 1.2 | | 14.3 | | NA | | NA | | 234 | | 0 | | 0 | | 0 | | 0 | | 0 | | 0 | | 0 | | 0 | |
| P5785 | 1 | | 17 | | MM EMERGENCY DEPT | | | 1 | | 2 | | 620 | | 2 | | 0 | | 1.1 | | 12 | | NA | | NA | | 172 | | 0 | | 0 | | 0 | | 1  (CTPA) | | 0 | | 0 | | 0 | | 0 | |
| P4269 | 1 | | 13 | | MM EMERGENCY DEPT | | | 1 | | 4 | | 128000 | | 2 | | 2 | | 2 | | 23.3 | | 38 | | 2.4 | | 34 | | 0 | | 0 | | 1  (CSVT) | | 1  (head MRI or CT) | | 1 | | 1 | | 0 | | 0 | |

APTT, activated partial thromboplastin time; B-ALL, B lymphoblastic leukemia; CART, chimeric antigen receptor T-cell therapy; CSVT, cerebral sinus venous thrombosis; CT, computed tomography; CTPA, computed tomography pulmonary angiography; DIC, disseminated intravascular coagulation; DVT, deep venous thrombosis; HL, Hodgkin’s lymphoma; IJV, internal jugular vein; INR, International Normalized Ratio; ISTH, International Society on Thrombosis and Hemostasis; KD, Kawasaki disease; MIS-C, multisystem inflammatory syndrome-children; MRI, magnetic resonance imaging; PE, pulmonary embolism; PERC, pulmonary embolism rule-out criteria; PT, prothrombin time; SCT, stem cell transplant; sJIA, systemic juvenile idiopathic arthritis; US, ultrasound; VTE, venous thromboembolism.

Collection department abbreviated: MM, McMaster University Medical Centre; PICU, pediatric intensive care unit; 3B2 HEM/ONC, hematology/ oncology inpatient ward; 3Y1 SURGERY, surgical inpatient ward; 3B1 EAT DISORDERS, outpatient eating disorder clinic; 3C MEDICINE, inpatient medical ward; SJHK URGENT CARE, St. Joseph’s Healthcare urgent care center emergency department; IR DEP, outpatient interventional radiology clinic; LAB SCC, outpatient laboratory; 3Z2 MEDICINE, inpatient pediatric medical ward; JH EMERGENCY DEPT, Juravinski Hospital emergency department; PED HEMATOLOGY, inpatient pediatric hematology unit; HGH, Hamilton General Hospital; SJHC 1; St. Joseph’s Healthcare emergency department; 3Z2 MEDICINE, inpatient medical ward; NICU, neonatal intensive care unit; OR, operation room; SJHC 3 SPEC CARE NURS, St. Joseph’s Healthcare inpatient nursery; PED ONC TMNT, outpatient pediatric oncology treatment room.

**Table S2.** **Summary of information on the adult patients who had D-dimer tests.** Laboratory results were obtained from historical records, with information on ages and sex. Diagnoses were obtained by review of electronic medical records. Coding for the reasons of D-dimer testing are as follows: (1, deep venous thrombosis; 2, pulmonary embolism; 3, arterial thrombosis; 4, disseminated intravascular coagulation; 5, chimeric antigen receptor therapy; 6, COVID-19 infection; 7, inflammatory conditions; 8, other venous thromboembolism; 9, other causes; 10, venous thromboembolism assessment; 0, unknown reason). Abbreviations of thrombosis diagnosed are at the bottom of the table.

| Code | sex 0=F 1=M | Age (yrs) | collection department | Alive on discharge  0=no 1=yes | Reason of D-dimer testing | first D-dimer level (µg/FEU) | D-dimer elevated  1=no, 2=yes | number of D-dimer repeats | INR | PT (sec) | APTT (sec) | Fibrinogen level (g/l) | lowest platelet count (x109/L) | One or more ISTH DIC scores recorded on chart 0=no, 1=yes | VTE pretest probability score done 0=no, 1=yes (specify) | acute thrombosis diagnosed 0=no  1=yes (specify) | imaging done 0=no 1=yes (specify) | hepatic impairment 0=no 1=yes | renal impairment 0=no 1=yes | recent surgery 0=no 1=yes | malignancy 0=no  1=yes (specify) |
| --- | --- | --- | --- | --- | --- | --- | --- | --- | --- | --- | --- | --- | --- | --- | --- | --- | --- | --- | --- | --- | --- |
| P3 | 0 | 70 | HGH EMERGENCY DEPT JH | 1 | 3 | 423 | 1 | 0 | 1 | 11 | 32 | NA | 204 | 0 | 0 | 0 | 0 | 0 | 0 | 0 | 0 |
| P192 | 1 | 60 | JH B4 HEMATOLOGY | 1 | 4 | 3220 | 2 | 3 | 1.1 | 13.4 | 35 | 6.9 | 15 | 0 | 0 | 0 | 0 | 0 | 0 | 0 | 1 |
| P381 | 1 | 72 | SJHC 1 EMERGENCY | 1 | 2 | 1910 | 2 | 0 | NA | NA | NA | NA | 189 | 0 | 0 | 0 | 1 (CTPA) | 0 | 0 | 0 | 0 |
| P190 | 1 | 75 | JH C4 HEMATOLOGY | 0 | 4 | 11200 | 2 | 6 | 1.4 | 16.3 | 31 | 7.2 | 63 | 0 | 0 | 0 | 0 | 0 | 0 | 0 | 1 |
| P193 | 1 | 75 | JH F5 MEDICINE | 1 | 1 | 1580 | 2 | 0 | 1.2 | 13.9 | NA | NA | 194 | 0 | 0 | 0 | 0 | 0 | 0 | 0 | 0 |
| P386 | 1 | 21 | SJHC 1 EMERGENCY | 1 | 2 | 988 | 2 | 0 | NA | NA | NA | NA | 250 | 0 | 1 (YEARS) | 0 | 0 | 0 | 0 | 0 | 0 |
| P7 | 0 | 86 | HGH EMERGENCY DEPT | 1 | 3 | 388 | 1 | 0 | 1.3 | 15.3 | NA | NA | 211 | 0 | 0 | 0 | 0 | 0 | 0 | 0 | 1 |
| P9 | 0 | 41 | SJHK URGENT CARE | 1 | 2 | 1580 | 2 | 0 | NA | NA | NA | NA | 316 | 0 | 0 | 0 | 1 (CTPA) | 0 | 0 | 0 | 0 |
| P384 | 1 | 67 | SJHC 1 EMERGENCY | 1 | 2 | 1680 | 2 | 0 | 1 | 11.2 | NA | NA | 534 | 0 | 1 (Wells) | 0 | 0 | 0 | 0 | 0 | 1 |
| P197 | 0 | 43 | JH EMERGENCY DEPT | 1 | 3 | 259 | 1 | 0 | 1.1 | 13 | NA | NA | 337 | 0 | 0 | 0 | 0 | 0 | 0 | 0 | 0 |
| P385 | 0 | 41 | SJHC 1 EMERGENCY | 1 | 3 | 247 | 1 | 0 | NA | NA | NA | NA | 283 | 0 | 0 | 0 | 0 | 0 | 0 | 0 | 0 |
| P14 | 1 | 87 | HGH EMERGENCY DEPT | 1 | 1 | 1510 | 2 | 0 | NA | NA | NA | NA | 185 | 0 | 0 | 0 | 1 (US) | 0 | 0 | 0 | 0 |
| P198 | 1 | 69 | JH EMERGENCY DEPT | 1 | 2 | 2260 | 2 | 0 | 1.4 | 15.8 | NA | NA | 217 | 0 | 0 | 0 | 1 (CTPA) | 0 | 1 | 0 | 1 |
| P8 | 1 | 64 | HGH EMERGENCY DEPT | 1 | 3 | 868 | 2 | 0 | 1.1 | 12.7 | NA | NA | 206 | 0 | 0 | 0 | 0 | 0 | 0 | 0 | 0 |
| P383 | 1 | 84 | SJHC 1 EMERGENCY | 1 | 2 | 5310 | 2 | 0 | 1.1 | 12.5 | NA | NA | 206 | 0 | 0 | 0 | 1 (CTPA) | 0 | 0 | 0 | 1 |
| P387 | 0 | 53 | SJHC 1 EMERGENCY | 1 | 1 | 407 | 1 | 0 | NA | NA | NA | NA | 281 | 0 | 0 | 0 | 0 | 0 | 0 | 0 | 0 |
| P15 | 1 | 33 | HGH EMERGENCY DEPT | 1 | 1 | 215 | 1 | 0 | 1.1 | 12.3 | NA | NA | 268 | 0 | 0 | 0 | 1 (US) | 0 | 0 | 0 | 0 |
| P199 | 0 | 69 | JH EMERGENCY DEPT | 1 | 1 | 488 | 1 | 0 | NA | NA | NA | NA | 248 | 0 | 1 (Wells & YEARS) | 0 | 0 | 0 | 0 | 1 | 0 |
| P201 | 0 | 79 | JH EMERGENCY DEPT | 1 | 2 | 857 | 2 | 0 | 1.2 | 13.8 | NA | NA | 73 | 0 | 0 | 0 | 1 (CTPA) | 0 | 1 | 0 | 1 |
| P13 | 1 | 67 | HGH ICU WEST | 0 | 3 | 14900 | 2 | 1 | 2.1 | 23.8 | NA | 3.7 | 85 | 0 | 0 | 1 (STEMI) | 0 | 1 | 1 | 1 | 0 |
| P21 | 0 | 53 | SJHK URGENT CARE | 1 | 3 | 215 | 1 | 0 | NA | NA | NA | NA | 199 | 0 | 0 | 0 | 0 | 0 | 0 | 0 | 0 |
| P203 | 0 | 48 | JH EMERGENCY DEPT | 1 | 3 | 288 | 1 | 0 | 1 | 11.1 | NA | NA | 217 | 0 | 0 | 0 | 0 | 0 | 0 | 0 | 0 |
| P23 | 1 | 79 | HGH EMERGENCY DEPT | 1 | 2 | 12300 | 2 | 0 | 1.1 | 12.6 | NA | NA | 181 | 0 | 0 | 0 | 1 (CTPA) | 0 | 1 | 0 | 1 |
| P27 | 1 | 63 | SJHK URGENT CARE | 1 | 3 | 360 | 1 | 0 | NA | NA | NA | NA | 235 | 0 | 0 | 0 | 0 | 0 | 0 | 0 | 0 |
| P22 | 1 | 82 | HGH EMERGENCY DEPT | 1 | 3 | 758 | 2 | 0 | 1.1 | 12.9 | NA | NA | 324 | 0 | 0 | 0 | 0 | 0 | 0 | 0 | 0 |
| P350 | 0 | 80 | WEC URGENT CARE CENTRE | 1 | 1 | 963 | 1 | 0 | NA | NA | NA | NA | 213 | 0 | 0 | 0 | 1 (US) | 0 | 0 | 0 | 0 |
| P390 | 1 | 63 | SJHC 1 INTENSIVE CARE | 1 | 1 | 844 | 2 | 0 | 1.2 | 14.1 | 34 | 1.6 | 62 | 0 | 0 | 0 | 0 | 1 | 0 | 0 | 0 |
| P32 | 0 | 22 | SJHK URGENT CARE | 1 | 2 | 367 | 1 | 0 | NA | NA | NA | NA | 132 | 0 | 0 | 0 | 0 | 0 | 0 | 0 | 0 |
| P392 | 1 | 73 | SJHC 1 EMERGENCY | 1 | 3 | 497 | 1 | 0 | NA | NA | NA | NA | 279 | 0 | 0 | 0 | 0 | 0 | 0 | 0 | 0 |
| P208 | 0 | 20 | JH EMERGENCY DEPT | 1 | 2 | 489 | 1 | 0 | NA | NA | NA | NA | 268 | 0 | 0 | 0 | 0 | 0 | 0 | 0 | 0 |
| P393 | 1 | 41 | SJHC 1 EMERGENCY | 1 | 1 | 843 | 2 | 0 | NA | NA | NA | NA | 248 | 0 | 0 | 0 | 1 (US) | 0 | 0 | 0 | 0 |
| P207 | 0 | 32 | JH EMERGENCY DEPT | 1 | 2 | 1080 | 2 | 0 | 0.9 | 10.9 | NA | NA | 217 | 0 | 0 | 0 | 1 (NM VQ scan for PE and US for DVT) | 0 | 0 | 0 | 0 |
| P394 | 1 | 25 | SJHC 1 EMERGENCY | 1 | 2 | 514 | 2 | 0 | NA | NA | NA | NA | 256 | 0 | 0 | 0 | 1 (CTPA) | 0 | 0 | 0 | 0 |
| P33 | 0 | 67 | HGH EMERGENCY DEPT | 1 | 1 | 1200 | 2 | 0 | 1.1 | 12.7 | NA | NA | 299 | 0 | 0 | 0 | 1 (US) | 0 | 0 | 0 | 0 |
| P396 | 0 | 52 | SJHC 1 EMERGENCY | 1 | 2 | 410 | 1 | 0 | NA | NA | NA | NA | 327 | 0 | 0 | 0 | 0 | 0 | 0 | 0 | 0 |
| P35 | 0 | 31 | HGH EMERGENCY DEPT | 1 | 2 | 438 | 1 | 0 | NA | NA | NA | NA | 239 | 0 | 0 | 0 | 0 | 0 | 0 | 0 | 0 |
| P36 | 1 | 53 | HGH EMERGENCY DEPT | 1 | 1 | 375 | 1 | 0 | 1 | 11.4 | 43 | 3.9 | 103 | 0 | 0 | 0 | 0 | 0 | 0 | 0 | 0 |
| P37 | 1 | 80 | HGH EMERGENCY DEPT | 1 | 3 | 304 | 1 | 0 | 1.1 | 12.6 | NA | NA | 185 | 0 | 0 | 1 (NSTEMI) | 0 | 0 | 0 | 0 | 0 |
| P210 | 1 | 28 | JH EMERGENCY DEPT | 1 | 2 | 881 | 2 | 0 | NA | NA | NA | NA | 272 | 0 | 1 (Wells & YEARS) | 0 | 0 | 0 | 0 | 0 | 0 |
| P399 | 1 | 63 | SJHC 1 EMERGENCY | 1 | 1 | 3300 | 2 | 0 | NA | NA | NA | NA | 364 | 0 | 0 | 0 | 1 (US) | 0 | 0 | 0 | 0 |
| P400 | 1 | 43 | SJHC 1 EMERGENCY | 1 | 1 | 633 | 2 | 0 | NA | NA | NA | NA | 197 | 0 | 0 | 0 | 1 (US) | 0 | 0 | 0 | 0 |
| P214 | 0 | 34 | JH EMERGENCY DEPT | 1 | 1 | 309 | 1 | 0 | NA | NA | NA | NA | 321 | 0 | 1 (Wells) | 0 | 0 | 0 | 0 | 0 | 0 |
| P39 | 0 | 63 | HGH EMERGENCY DEPT | 1 | 2 | 7200 | 2 | 0 | 1.3 | 15.6 | 28 | NA | 132 | 0 | 0 | 0 | 1 (CTPA) | 0 | 0 | 0 | 0 |
| P353 | 1 | 27 | MM LAB SCC | 1 | 9 (hemophilia follow up) | 862 | 2 | 0 | NA | NA | 124 | NA | NA | 0 | 0 | 0 | 0 | 0 | 0 | 0 | 0 |
| P43 | 1 | 53 | HGH EMERGENCY DEPT | 1 | 3 | 482 | 1 | 0 | 1 | 11 | NA | NA | 223 | 0 | 0 | 0 | 0 | 0 | 0 | 0 | 0 |
| P216 | 1 | 67 | JH ODS | 1 | 5  (triple hit lymphoma) | 3660 | 2 | 6 | 1.3 | 15.5 | 28 | 3.2 | 5 | 0 | 0 | 0 | 0 | 0 | 1 | 0 | 1 |
| P217 | 1 | 72 | JH ODS | 1 | 5  (DLBCL) | 1670 | 2 | 3 | 1.1 | 13 | 42 | 2 | 75 | 0 | 0 | 0 | 0 | 0 | 0 | 0 | 1 |
| P44 | 1 | 85 | HGH EMERGENCY DEPT | 1 | 2 | 1110 | 2 | 0 | 1.2 | 14.1 | NA | NA | 181 | 0 | 0 | 0 | 1 (CTPA) | 0 | 1 | 0 | 1 |
| P218 | 0 | 64 | JCC LAB SCC | 1 | 0  (multiple myeloma) | 218 | 1 | 0 | 0.9 | 10.2 | 42 | 2.6 | 303 | 0 | 0 | 0 | 0 | 0 | 0 | 0 | 1 |
| P219 | 1 | 59 | JH LAB SCC | 1 | 1 | 500 | 2 | 0 | NA | NA | NA | NA | NA | 0 | 0 | 0 | 1 (US) | 0 | 0 | 0 | 0 |
| P45 | 1 | 53 | HGH EMERGENCY DEPT | 1 | 3 | 215 | 1 | 0 | 1 | 11.8 | NA | NA | 154 | 0 | 1 (Wells) | 0 | 0 | 0 | 0 | 0 | 0 |
| P406 | 0 | 80 | SJHK URGENT CARE | 1 | 1 | 903 | 2 | 0 | NA | NA | NA | NA | 306 | 0 | 0 | 0 | 1 (US) | 0 | 0 | 0 | 0 |
| P404 | 0 | 64 | SJHC FONTBONNE LAB | 1 | 1 | 522 | 2 | 0 | NA | NA | NA | NA | 283 | 0 | 0 | 0 | 0 | 0 | 0 | 0 | 0 |
| P47 | 1 | 79 | HGH EMERGENCY DEPT | 1 | 1 | 1360 | 2 | 0 | 1.1 | 12.7 | NA | NA | 139 | 0 | 1 (Wells) | 0 | 1 (CTPA) | 0 | 0 | 1 | 0 |
| P209 | 0 | 64 | JH EMERGENCY DEPT | 1 | 1 | 381 | 1 | 0 | 0.9 | 10.8 | NA | NA | 264 | 0 | 0 | 0 | 0 | 0 | 0 | 0 | 0 |
| P380 | 0 | 51 | SJHC 1 INTENSIVE CARE | 0 | 4 | 6650 | 2 | 0 | 2.8 | 33.6 | 64 | 1.3 | 6 | 0 | 0 | 0 | 0 | 1 | 1 | 0 | 0 |
| P408 | 0 | 60 | SJHC 1 EMERGENCY | 1 | 2 | 317 | 1 | 0 | NA | NA | NA | NA | 240 | 0 | 0 | 0 | 0 | 0 | 0 | 1 | 0 |
| P409 | 0 | 57 | SJHC 1 EMERGENCY | 1 | 2 | 357 | 1 | 0 | NA | NA | NA | NA | 212 | 0 | 0 | 0 | 0 | 0 | 0 | 0 | 0 |
| P410 | 0 | 64 | SJHC 1 EMERGENCY | 1 | 1 | 1630 | 2 | 0 | NA | NA | NA | NA | 256 | 0 | 0 | 0 | 1 (US) | 0 | 0 | 0 | 0 |
| P411 | 0 | 56 | SJHC 1 EMERGENCY | 1 | 2 | 1370 | 2 | 0 | NA | NA | NA | NA | 321 | 0 | 0 | 0 | 1 (CT) | 0 | 0 | 0 | 1 |
| P49 | 1 | 48 | HGH EMERGENCY DEPT | 1 | 1 | 579 | 2 | 0 | 0.9 | 10.5 | NA | NA | 306 | 0 | 1 (Wells) | 0 | 1 (US) | 0 | 0 | 0 | 0 |
| P413 | 0 | 59 | SJHC 1 EMERGENCY | 1 | 2 | 476 | 1 | 0 | NA | NA | NA | NA | 307 | 0 | 0 | 0 | 0 | 0 | 0 | 0 | 0 |
| P414 | 1 | 32 | SJHC 1 EMERGENCY | 1 | 3 | 2330 | 2 | 0 | NA | NA | NA | NA | 339 | 0 | 0 | 0 | 0 | 0 | 0 | 0 | 0 |
| P415 | 0 | 19 | SJHK URGENT CARE | 1 | 2 | 533 | 2 | 0 | NA | NA | NA | NA | 260 | 0 | 0 | 0 | 0 | 0 | 0 | 0 | 0 |
| P416 | 0 | 37 | SJHK URGENT CARE | 1 | 3 | 257 | 1 | 0 | NA | NA | NA | NA | 213 | 0 | 0 | 0 | 0 | 0 | 0 | 0 | 0 |
| P222 | 0 | 35 | JH EMERGENCY DEPT | 1 | 1 | 499 | 1 | 0 | 1 | 12.3 | 33 | NA | 207 | 0 | 1 (Wells) | 0 | 1 (CT) | 0 | 0 | 0 | 0 |
| P417 | 0 | 79 | SJHC 1 EMERGENCY | 1 | 2 | 1300 | 1 | 0 | NA | NA | NA | NA | 310 | 0 | 0 | 0 | 1 (CTPA) | 0 | 0 | 0 | 1 |
| P419 | 1 | 64 | SJHC 1 EMERGENCY | 1 | 2 | 312 | 1 | 0 | NA | NA | NA | NA | NA | 0 | 1 (Wells & PERC) | 0 | 0 | 0 | 0 | 0 | 0 |
| P422 | 0 | 29 | SJHC 1 EMERGENCY | 1 | 2 | 215 | 1 | 0 | NA | NA | NA | NA | 133 | 0 | 0 | 0 | 0 | 0 | 0 | 0 | 0 |
| P225 | 1 | 43 | JH EMERGENCY DEPT | 1 | 3 | 215 | 1 | 0 | 1 | 11.9 | 29 | NA | 192 | 0 | 0 | 0 | 0 | 0 | 0 | 0 | 0 |
| P53 | 0 | 86 | HGH EMERGENCY DEPT | 1 | 3 | 588 | 1 | 0 | 1 | 11.8 | NA | NA | 276 | 0 | 0 | 0 | 0 | 0 | 0 | 0 | 0 |
| P54 | 1 | 44 | HGH EMERGENCY DEPT | 1 | 3 | 215 | 1 | 0 | 1.2 | 14.2 | NA | NA | 287 | 0 | 0 | 0 | 0 | 0 | 0 | 0 | 0 |
| P424 | 0 | 32 | SJHC 1 EMERGENCY | 1 | 2 | 347 | 1 | 0 | NA | NA | NA | NA | 240 | 0 | 0 | 0 | 0 | 0 | 0 | 0 | 0 |
| P55 | 1 | 85 | HGH EMERGENCY DEPT | 1 | 2 | 1480 | 2 | 0 | 1.3 | 14.8 | NA | NA | 182 | 0 | 0 | 0 | 1 (CTPA) | 0 | 0 | 0 | 0 |
| P426 | 1 | 65 | SJHC 1 EMERGENCY | 1 | 2 | 332 | 1 | 0 | NA | NA | NA | NA | 166 | 0 | 0 | 0 | 0 | 0 | 0 | 0 | 0 |
| P427 | 0 | 78 | SJHC 1 EMERGENCY | 1 | 1 | 5790 | 2 | 0 | NA | NA | NA | NA | 236 | 0 | 0 | 1 (DVT confirmed by US) | 1 (US) | 0 | 1 | 0 | 0 |
| P354 | 0 | 90 | WEC URGENT CARE CENTRE | 1 | 1 | 1340 | 2 | 0 | NA | NA | NA | NA | 462 | 0 | 0 | 0 | 1 (US) | 0 | 1 | 0 | 0 |
| P230 | 0 | 87 | JH EMERGENCY DEPT | 1 | 1 | 2370 | 2 | 0 | 1 | 11.7 | NA | NA | 136 | 0 | 0 | 0 | 1 (US & CTPA) | 0 | 1 | 0 | 1 |
| P428 | 1 | 67 | SJHK URGENT CARE | 1 | 3 | 2270 | 2 | 0 | NA | NA | NA | NA | 378 | 0 | 0 | 0 | 0 | 0 | 0 | 1 | 0 |
| P429 | 1 | 72 | SJHK URGENT CARE | 1 | 2 | 1020 | 2 | 0 | NA | NA | NA | NA | 454 | 0 | 1 (YEARS) | 0 | 1 (CTPA) | 0 | 0 | 0 | 0 |
| P231 | 0 | 28 | JH EMERGENCY DEPT | 1 | 2 | 215 | 1 | 0 | 1.1 | 13.2 | NA | NA | 355 | 0 | 0 | 0 | 0 | 0 | 0 | 0 | 0 |
| P232 | 0 | 82 | JH EMERGENCY DEPT | 1 | 2 | 6840 | 2 | 0 | 1.4 | 16.8 | 29 | NA | 213 | 0 | 0 | 0 | 0 | 0 | 1 | 0 | 0 |
| P430 | 0 | 76 | SJHC 4D CTU | 1 | 2 | 618 | 2 | 2 | NA | NA | NA | NA | 198 | 0 | 0 | 0 | 0 | 0 | 1 | 0 | 0 |
| P431 | 1 | 30 | SJHC 1 EMERGENCY | 1 | 3 | 215 | 1 | 0 | NA | NA | NA | NA | 272 | 0 | 0 | 0 | 0 | 1 | 0 | 0 | 0 |
| P234 | 1 | 64 | JH EMERGENCY DEPT | 1 | 2 | 21100 | 2 | 0 | 1.2 | 14.4 | 33 | NA | 179 | 0 | 0 | 1 (PE confirmed by CTPA) | 1 (CTPA) | 0 | 0 | 1 | 1 |
| P235 | 1 | 72 | JH EMERGENCY DEPT | 0 | 2 | 2920 | 2 | 0 | NA | NA | NA | NA | 241 | 0 | 1 (Wells) | 0 | 1 (Chest XR & US for DVT) | 0 | 0 | 0 | 1 |
| P233 | 1 | 89 | JH EMERGENCY DEPT | 1 | 1 | 5560 | 2 | 0 | 1.2 | 13.6 | NA | NA | 195 | 0 | 0 | 0 | 1 (CTPA) | 0 | 1 | 0 | 0 |
| P60 | 1 | 82 | HGH EMERGENCY DEPT | 1 | 3 | 297 | 1 | 0 | 1 | 12 | NA | NA | 215 | 0 | 0 | 0 | 0 | 0 | 0 | 0 | 0 |
| P61 | 0 | 58 | HGH EMERGENCY DEPT | 1 | 1 | 240 | 1 | 0 | 1 | 11.6 | NA | NA | 325 | 0 | 0 | 0 | 0 | 0 | 0 | 0 | 0 |
| P432 | 1 | 57 | SJHC MSAU SSDU | 1 | 1 | 6470 | 2 | 0 | 1.2 | 14.3 | 26 | NA | 67 | 0 | 0 | 0 | 0 | 1 | 0 | 0 | 0 |
| P237 | 0 | 34 | JH EMERGENCY DEPT | 1 | 2 | 345 | 1 | 0 | 1 | 12.2 | NA | NA | 265 | 0 | 0 | 0 | 1 (CTPA) | 0 | 0 | 0 | 1 |
| P62 | 0 | 63 | HGH EMERGENCY DEPT | 1 | 2 | 359 | 1 | 0 | 1 | 12 | NA | NA | 291 | 0 | 0 | 0 | 0 | 0 | 1 | 0 | 0 |
| P435 | 0 | 63 | SJHC FONTBONNE LAB | 1 | 9  (follow up VTE) | 447 | 1 | 0 | NA | NA | NA | NA | NA | 0 | 0 | 0 | 0 | 0 | 0 | 0 | 0 |
| P238 | 1 | 56 | JH EMERGENCY DEPT | 1 | 1 | 320 | 1 | 0 | NA | NA | NA | NA | 392 | 0 | 0 | 0 | 0 | 0 | 0 | 0 | 0 |
| P240 | 1 | 67 | JH EMERGENCY DEPT | 1 | 2 | 218 | 1 | 0 | 0.9 | 11.1 | NA | NA | 171 | 0 | 0 | 0 | 0 | 0 | 0 | 0 | 0 |
| P437 | 1 | 73 | SJHC 1 EMERGENCY | 1 | 2 | 1520 | 2 | 0 | NA | NA | NA | NA | 239 | 0 | 0 | 0 | 1 (CTPA) | 0 | 0 | 0 | 0 |
| P58 | 1 | 64 | HGH EMERGENCY DEPT | 1 | 4 | 642 | 2 | 0 | 1.2 | 13.1 | NA | 5.9 | 19 | 0 | 0 | 0 | 0 | 0 | 0 | 0 | 0 |
| P439 | 1 | 87 | SJHC 1 EMERGENCY | 1 | 1 | 1200 | 2 | 0 | NA | NA | NA | NA | NA | 0 | 0 | 0 | 1 (US) | 0 | 1 | 0 | 0 |
| P441 | 1 | 77 | SJHC FONTBONNE LAB | 1 | 4 | 328 | 1 | 0 | 2 | 23.1 | 41 | 2.5 | 82 | 0 | 0 | 0 | 0 | 0 | 0 | 0 | 0 |
| P16 | 1 | 75 | HGH ICU WEST | 0 | 3 | 296 | 1 | 0 | 2.6 | 30.8 | 36 | 5.7 | 176 | 0 | 0 | 1 (NSTEMI) | 0 | 1 | 1 | 1 | 0 |
| P440 | 0 | 42 | SJHK URGENT CARE | 1 | 1 | 342 | 1 | 0 | NA | NA | NA | NA | 261 | 0 | 0 | 0 | 1 (US) | 0 | 0 | 0 | 1 |
| P443 | 0 | 26 | SJHC 1 EMERGENCY | 1 | 2 | 1790 | 2 | 0 | 1 | 12 | NA | NA | 238 | 0 | 0 | 0 | 1 (CTPA) | 0 | 1 | 1 | 0 |
| P68 | 0 | 47 | HGH EMERGENCY DEPT | 1 | 1 | 364 | 1 | 0 | NA | NA | NA | NA | 345 | 0 | 0 | 0 | 1 (US) | 0 | 0 | 0 | 0 |
| P445 | 0 | 63 | SJHK URGENT CARE | 1 | 3 | 4640 | 2 | 0 | NA | NA | NA | NA | 230 | 0 | 0 | 0 | 0 | 0 | 0 | 0 | 1 |
| P50 | 0 | 52 | HGH 5W MEDICINE | 1 | 4 | 1140 | 2 | 1 | 1.6 | 18.5 | 40 | 8.4 | 239 | 0 | 0 | 0 | 0 | 0 | 1 | 0 | 0 |
| P249 | 0 | 29 | JH EMERGENCY DEPT | 1 | 2 | 283 | 1 | 0 | NA | NA | NA | NA | 293 | 0 | 0 | 0 | 0 | 0 | 0 | 0 | 0 |
| P70 | 1 | 63 | HGH EMERGENCY DEPT | 1 | 2 | 281 | 1 | 0 | 1.1 | 12.2 | NA | NA | 299 | 0 | 1 (Wells & PERC) | 0 | 0 | 0 | 0 | 0 | 0 |
| P388 | 1 | 65 | SJHC 5MG CTU | 1 | 2 | 2230 | 2 | 0 | 1.1 | 12.8 | NA | NA | 194 | 0 | 0 | 0 | 1 (CTPA) | 0 | 1 | 0 | 0 |
| P250 | 0 | 61 | JH C3 ONCOLOGY/GI | 1 | 2 | 297 | 1 | 0 | 1 | 11.8 | NA | NA | 96 | 0 | 0 | 0 | 0 | 0 | 0 | 0 | 1 |
| P446 | 0 | 45 | SJHC 1 EMERGENCY | 1 | 2 | 502 | 2 | 0 | NA | NA | NA | NA | 352 | 0 | 0 | 0 | 0 | 0 | 0 | 1 | 0 |
| P242 | 0 | 64 | JH EMERGENCY DEPT | 1 | 2 | 4120 | 2 | 0 | 0.9 | 10.4 | NA | NA | 107 | 0 | 0 | 0 | 1 (CTPA) | 0 | 1 | 0 | 1 |
| P251 | 1 | 73 | JH EMERGENCY DEPT | 0 | 2 | 1350 | 2 | 0 | 1.2 | 14.1 | NA | NA | 188 | 0 | 0 | 0 | 0 | 0 | 1 | 0 | 0 |
| P246 | 1 | 52 | JH ICU LEVEL 1 | 1 | 2 | 471 | 1 | 0 | 0.8 | 9.8 | NA | NA | 160 | 0 | 0 | 0 | 0 | 0 | 0 | 0 | 0 |
| P448 | 1 | 28 | SJHK URGENT CARE | 1 | 2 | 1550 | 2 | 0 | NA | NA | NA | NA | 233 | 0 | 0 | 0 | 0 | 0 | 0 | 0 | 0 |
| P69 | 1 | 50 | HGH EMERGENCY DEPT | 1 | 3 | 243 | 1 | 0 | 1 | 10.9 | NA | NA | 304 | 0 | 0 | 0 | 0 | 0 | 0 | 0 | 0 |
| P449 | 0 | 37 | SJHC 1 EMERGENCY | 1 | 1 | 1640 | 2 | 0 | NA | NA | NA | NA | 276 | 0 | 0 | 0 | 0 | 0 | 0 | 1 | 0 |
| P450 | 0 | 34 | SJHC 1 EMERGENCY | 1 | 1 | 373 | 1 | 0 | NA | NA | NA | NA | 227 | 0 | 0 | 0 | 0 | 0 | 0 | 0 | 0 |
| P75 | 0 | 62 | HGH EMERGENCY DEPT | 1 | 2 | 3770 | 2 | 0 | 1 | 11.6 | NA | NA | 315 | 0 | 0 | 0 | 1 (CTPA) | 0 | 0 | 0 | 0 |
| P63 | 1 | 78 | HGH 4W VASCULAR | 1 | 3 | 1210 | 2 | 0 | 2.4 | 27.6 | NA | NA | 155 | 0 | 0 | 0 | 0 | 0 | 1 | 0 | 0 |
| P256 | 0 | 58 | JH EMERGENCY DEPT | 1 | 2 | 265 | 1 | 0 | NA | NA | NA | NA | 290 | 0 | 0 | 0 | 0 | 0 | 0 | 0 | 0 |
| P67 | 1 | 84 | HGH 4W CARDIOLOGY | 1 | 2 | 1710 | 2 | 0 | 3 | 34.2 | NA | NA | 207 | 0 | 0 | 0 | 0 | 0 | 1 | 0 | 0 |
| P257 | 1 | 57 | JH EMERGENCY DEPT | 1 | 2 | 6090 | 2 | 0 | 1.2 | 14.5 | NA | NA | 599 | 0 | 0 | 0 | 1 (CTPA) | 0 | 0 | 0 | 0 |
| P76 | 0 | 62 | HGH EMERGENCY DEPT | 1 | 2 | 1470 | 2 | 0 | 1.3 | 15 | 32 | NA | 276 | 0 | 0 | 2 (confirmed by CTPA) | 1 (CTPA) | 0 | 0 | 1 | 0 |
| P254 | 0 | 86 | JH EMERGENCY DEPT | 1 | 2 | 374 | 1 | 0 | 1 | 12.2 | NA | NA | 262 | 0 | 0 | 0 | 0 | 0 | 1 | 0 | 0 |
| P258 | 0 | 66 | JH EMERGENCY DEPT | 1 | 2 | 1760 | 2 | 0 | 1.6 | 18.9 | NA | NA | 180 | 0 | 0 | 0 | 1 (CTPA) | 0 | 1 | 0 | 0 |
| P81 | 0 | 47 | HGH EMERGENCY DEPT | 1 | 3 | 290 | 1 | 0 | 1 | 11 | NA | NA | 244 | 0 | 0 | 0 | 0 | 0 | 0 | 0 | 0 |
| P82 | 0 | 67 | HGH EMERGENCY DEPT | 1 | 3 | 876 | 2 | 0 | 1 | 11.4 | NA | na | 219 | 0 | 0 | 0 | 0 | 0 | 0 | 0 | 0 |
| P83 | 0 | 67 | HGH EMERGENCY DEPT | 1 | 3 | 360 | 1 | 0 | 1 | 11.2 | NA | NA | 205 | 0 | 0 | 0 | 0 | 0 | 0 | 0 | 0 |
| P452 | 1 | 69 | SJHC 1 EMERGENCY | 1 | 2 | 320 | 1 | 0 | NA | NA | NA | NA | 169 | 0 | 0 | 0 | 0 | 0 | 1 | 0 | 1 |
| P5 | 1 | 29 | HGH ICU EAST/SOUTH | 1 | 4 | 6520 | 2 | 21 | 1 | 11.4 | 26 | 5.1 | 39 | 0 | 0 | 0 | 0 | 0 | 0 | 0 | 0 |
| P453 | 0 | 88 | SJHC 5MG CTU | 1 | 3 | 1100 | 2 | 0 | 1.1 | 13.3 | NA | NA | 200 | 0 | 0 | 0 | 0 | 0 | 1 | 0 | 0 |
| P447 | 1 | 71 | SJHC 1 EMERGENCY | 1 | 2 | 1200 | 2 | 0 | 2.6 | 30.3 | NA | NA | 159 | 0 | 0 | 0 | 0 | 0 | 0 | 0 | 0 |
| P85 | 1 | 88 | HGH EMERGENCY DEPT | 1 | 2 | 2200 | 2 | 0 | 1.4 | 16.4 | NA | NA | 220 | 0 | 0 | 1 (PE confirmed by CTPA) | 1 (CTPA) | 0 | 1 | 0 | 0 |
| P454 | 0 | 58 | SJHC T7 NEPHROLOGY | 1 | 1 | 13200 | 2 | 0 | 1.1 | 12.3 | 27 | 5.4 | 106 | 0 | 0 | 1 (DVT confirmed by US) | 1 (US) | 0 | 1 | 1 | 0 |
| P462 | 1 | 72 | SJHC 1 EMERGENCY | 1 | 0  (COPD) | 402 | 1 | 0 | NA | NA | NA | NA | 135 | 0 | 0 | 0 | 0 | 0 | 0 | 0 | 0 |
| P463 | 0 | 54 | SJHK URGENT CARE | 1 | 2 | 624 | 2 | 0 | NA | NA | NA | NA | 310 | 0 | 1 (Wells) | 0 | 0 | 0 | 0 | 0 | 0 |
| P266 | 1 | 61 | JH EMERGENCY DEPT | 1 | 2 | 522 | 2 | 0 | NA | NA | NA | NA | 122 | 0 | 0 | 0 | 1 (CTPA) | 0 | 0 | 0 | 1 |
| P86 | 0 | 55 | HGH EMERGENCY DEPT | 1 | 1 | 708 | 2 | 0 | NA | NA | NA | NA | 268 | 0 | 0 | 0 | 1 (US) | 0 | 0 | 0 | 0 |
| P464 | 0 | 76 | SJHC 1 EMERGENCY | 1 | 2 | 580 | 2 | 0 | NA | NA | NA | NA | 382 | 0 | 0 | 1 (PE confirmed by CTPA) | 1 (CTPA) | 0 | 0 | 0 | 0 |
| P263 | 1 | 64 | JH EMERGENCY DEPT | 1 | 2 | 912 | 2 | 0 | 1 | 11.3 | NA | NA | 212 | 0 | 0 | 1 (PE confirmed by NM VQ scan) | 1 (NM VQ scan) | 0 | 0 | 0 | 0 |
| P265 | 0 | 81 | JH EMERGENCY DEPT | 1 | 3 | 3960 | 2 | 0 | 1 | 11.5 | NA | NA | 165 | 0 | 0 | 0 | 0 | 0 | 0 | 0 | 0 |
| P465 | 0 | 60 | SJHC 1 EMERGENCY | 1 | 4 | 4260 | 2 | 0 | 1.4 | 16.7 | NA | NA | 124 | 0 | 0 | 0 | 0 | 0 | 1 | 0 | 0 |
| P267 | 0 | 84 | JH EMERGENCY DEPT | 1 | 1 | 742 | 2 | 0 | 1 | 12.1 | NA | NA | 307 | 0 | 0 | 0 | 0 | 0 | 0 | 1 | 0 |
| P73 | 1 | 57 | HGH 7S STROKE/NEURO | 1 | 3 | 534 | 2 | 0 | NA | NA | 34 | NA | 316 | 0 | 0 | 1 (ischemic stroke confirmed by MRI) | 1 (MRI Brain) | 0 | 0 | 0 | 0 |
| P52 | 1 | 83 | HGH 6W ORTHO | 1 | 1 | 999 | 2 | 0 | 1.4 | 16.8 | 33 | 3.4 | 357 | 0 | 0 | 1 (ischemic extremity confirmed by CTA) | 1 (lower extremity CTA) | 0 | 0 | 1 | 0 |
| P262 | 1 | 62 | JH ODS | 1 | 5  (tNHL) | 3870 | 2 | 22 | 1.2 | 13.8 | 26 | 5.3 | 77 | 0 | 0 | 0 | 0 | 0 | 0 | 0 | 1 |
| P272 | 1 | 71 | JH E3 MEDICINE | 1 | 2 | 629 | 2 | 0 | NA | NA | NA | NA | 190 | 0 | 1 (Wells) | 0 | 0 | 0 | 0 | 0 | 0 |
| P248 | 0 | 31 | JH EMERGENCY DEPT | 1 | 2 | 425 | 1 | 0 | 1 | 12.1 | NA | NA | 213 | 0 | 0 | 0 | 0 | 0 | 0 | 0 | 0 |
| P470 | 1 | 70 | SJHC 1 EMERGENCY | 1 | 2 | 502 | 2 | 0 | 1.1 | 12.4 | NA | NA | 167 | 0 | 0 | 0 | 0 | 0 | 0 | 0 | 0 |
| P471 | 0 | 78 | SJHC 1 EMERGENCY | 1 | 2 | 496 | 1 | 1 | 1 | 12.2 | NA | NA | 183 | 0 | 0 | 0 | 0 | 0 | 0 | 0 | 0 |
| P469 | 0 | 45 | SJHC MSAU H&N | 1 | 2 | 3960 | 2 | 0 | 1.1 | 12.9 | NA | NA | 335 | 0 | 0 | 0 | 0 | 0 | 0 | 1 | 1 |
| P473 | 0 | 47 | SJHC 1 EMERGENCY | 1 | 2 | 1500 | 2 | 0 | NA | NA | NA | NA | 317 | 0 | 0 | 0 | 1 (CTPA) | 0 | 0 | 0 | 0 |
| P474 | 0 | 74 | SJHC 1 EMERGENCY | 1 | 2 | 873 | 2 | 0 | NA | NA | NA | NA | 496 | 0 | 0 | 0 | 0 | 0 | 0 | 0 | 0 |
| P475 | 1 | 70 | SJHK URGENT CARE | 1 | 1 | 1400 | 2 | 0 | NA | NA | NA | NA | 166 | 0 | 1 (Wells) | 0 | 1 (US) | 0 | 0 | 0 | 0 |
| P89 | 0 | 72 | HGH EMERGENCY DEPT | 1 | 2 | 484 | 1 | 0 | 1.1 | 12.6 | 29 | NA | 265 | 0 | 0 | 1 (PE confirmed by CTPA) | 1 (CTPA) | 0 | 1 | 0 | 0 |
| P91 | 0 | 64 | HGH EMERGENCY DEPT | 1 | 1 | 394 | 1 | 0 | 1 | 11.9 | NA | NA | 278 | 0 | 0 | 0 | 0 | 0 | 0 | 0 | 0 |
| P477 | 0 | 36 | SJHK URGENT CARE | 1 | 3 | 215 | 1 | 0 | NA | NA | NA | NA | 223 | 0 | 0 | 0 | 0 | 0 | 0 | 0 | 0 |
| P479 | 0 | 18 | SJHK URGENT CARE | 1 | 2 | 556 | 2 | 0 | NA | NA | NA | NA | NA | 0 | 1 (YEARS) | 0 | 0 | 0 | 0 | 0 | 0 |
| P88 | 1 | 64 | HGH EMERGENCY DEPT | 1 | 2 | 1120 | 2 | 0 | 1.1 | 12.6 | NA | NA | 192 | 0 | 0 | 0 | 0 | 0 | 0 | 0 | 0 |
| P1 | 1 | 57 | HGH 6S SURG/TRAUMA | 0 | 9 (HLH) | 1030 | 2 | 0 | 1.4 | 16.1 | 61 | 4.6 | 20 | 0 | 0 | 0 | 0 | 0 | 0 | 1 | 1 |
| P476 | 1 | 62 | SJHC 1 INTENSIVE CARE | 1 | 2 | 952 | 2 | 0 | 1.3 | 14.6 | NA | 7.3 | 53 | 0 | 0 | 0 | 0 | 0 | 0 | 0 | 0 |
| P275 | 0 | 77 | JH EMERGENCY DEPT | 1 | 2 | 1480 | 2 | 1 | 1.2 | 13.8 | 31 | NA | 121 | 0 | 0 | 1 (segmental PE confirmed by CTPA) | 1 (CTPA & US) | 0 | 1 | 0 | 1 |
| P93 | 0 | 20 | HGH EMERGENCY DEPT | 1 | 3 | 215 | 1 | 1 | 1.2 | 13.7 | 31 | NA | 302 | 0 | 0 | 0 | 0 | 0 | 0 | 0 | 0 |
| P95 | 0 | 57 | HGH EMERGENCY DEPT | 1 | 2 | 390 | 1 | 2 | 1.1 | 12.5 | 29 | NA | 232 | 0 | 0 | 0 | 0 | 0 | 0 | 0 | 0 |
| P226 | 0 | 76 | JH E4 SURGERY | 1 | 9 (post operative) | 4440 | 2 | 0 | 1.2 | 14 | 28 | NA | 194 | 0 | 0 | 0 | 0 | 1 | 0 | 1 | 1 |
| P96 | 1 | 52 | HGH EMERGENCY DEPT | 1 | 1 | 850 | 2 | 0 | NA | NA | NA | NA | 146 | 0 | 0 | 0 | 1 (US) | 0 | 0 | 0 | 0 |
| P274 | 1 | 94 | JH EMERGENCY DEPT | 1 | 3 | 2060 | 2 | 0 | 1.2 | 13.5 | NA | NA | 132 | 0 | 0 | 0 | 0 | 0 | 1 | 0 | 0 |
| P97 | 0 | 38 | HGH EMERGENCY DEPT | 1 | 3 | 564 | 2 | 0 | 1 | 11.9 | NA | NA | 311 | 0 | 0 | 0 | 0 | 0 | 0 | 0 | 0 |
| P481 | 1 | 55 | SJHC 1 EMERGENCY | 1 | 1 | 4370 | 2 | 0 | NA | NA | NA | NA | 717 | 0 | 0 | 0 | 1 (US) | 1 | 0 | 0 | 1 |
| P482 | 0 | 66 | SJHC 1 EMERGENCY | 1 | 2 | 1380 | 2 | 0 | NA | NA | NA | NA | 241 | 0 | 1 (Wells & PERC) | 0 | 0 | 0 | 1 | 0 | 0 |
| P100 | 0 | 37 | GH EMERGENCY DEPT | 1 | 2 | 233 | 1 | 0 | NA | NA | NA | NA | 207 | 0 | 0 | 0 | 0 | 1 | 0 | 0 | 0 |
| P101 | 0 | 31 | HGH EMERGENCY DEPT | 1 | 2 | 1110 | 2 | 0 | 0.9 | 10.5 | NA | NA | 125 | 0 | 0 | 0 | 1 (US & CTPA) | 0 | 0 | 0 | 0 |
| P484 | 0 | 19 | SJHC 1 EMERGENCY | 1 | 2 | 343 | 1 | 0 | NA | NA | NA | NA | 305 | 0 | 0 | 0 | 0 | 0 | 0 | 0 | 0 |
| P485 | 1 | 52 | SJHC 1 EMERGENCY | 1 | 1 | 354 | 1 | 0 | NA | NA | NA | NA | 218 | 0 | 0 | 0 | 0 | 0 | 0 | 0 | 0 |
| P277 | 1 | 59 | JH EMERGENCY DEPT | 1 | 1 | 363 | 1 | 0 | 1 | 11.3 | NA | NA | 317 | 0 | 0 | 0 | 0 | 0 | 0 | 0 | 1 |
| P486 | 0 | 42 | SJHK URGENT CARE | 1 | 3 | 215 | 1 | 0 | NA | NA | NA | NA | 184 | 0 | 0 | 0 | 0 | 0 | 0 | 0 | 0 |
| P487 | 0 | 30 | SJHK URGENT CARE | 1 | 3 | 1040 | 2 | 0 | NA | NA | NA | NA | 336 | 0 | 0 | 0 | 0 | 0 | 0 | 0 | 0 |
| P489 | 1 | 60 | SJHC 1 EMERGENCY | 1 | 1 | 431 | 1 | 0 | NA | NA | NA | NA | 209 | 0 | 0 | 0 | 1 (US) | 0 | 0 | 0 | 0 |
| P103 | 0 | 63 | HGH EMERGENCY DEPT | 1 | 2 | 2280 | 2 | 0 | 1.5 | 17 | NA | NA | 152 | 0 | 0 | 1 (PE confirmed by CTPA) | 1 (CTPA) | 1 | 0 | 0 | 0 |
| P490 | 0 | 66 | SJHC 1 EMERGENCY | 1 | 2 | 317 | 1 | 0 | NA | NA | NA | NA | 382 | 0 | 0 | 0 | 0 | 0 | 0 | 0 | 0 |
| P491 | 1 | 67 | SJHC 1 EMERGENCY | 1 | 2 | 614 | 2 | 0 | NA | NA | NA | NA | 225 | 0 | 0 | 0 | 1 (CTPA) | 0 | 0 | 0 | 0 |
| P104 | 1 | 52 | HGH EMERGENCY DEPT | 1 | 2 | 298 | 1 | 0 | 1.1 | 12.5 | NA | NA | 260 | 0 | 0 | 0 | 0 | 0 | 0 | 0 | 0 |
| P492 | 1 | 74 | SJHK URGENT CARE | 1 | 1 | 824 | 2 | 0 | NA | NA | NA | NA | NA | 0 | 0 | 0 | 1 (US) | 0 | 0 | 0 | 0 |
| P362 | 0 | 46 | WEC URGENT CARE CENTRE | 1 | 1 | 443 | 1 | 0 | NA | NA | NA | NA | 225 | 0 | 1 (Wells) | 0 | 1 (US) | 0 | 0 | 0 | 0 |
| P493 | 0 | 18 | SJHC 1 EMERGENCY | 1 | 1 | 295 | 1 | 0 | NA | NA | NA | NA | 390 | 0 | 0 | 0 | 0 | 0 | 0 | 0 | 0 |
| P102 | 0 | 35 | HGH EMERGENCY DEPT | 1 | 2 | 245 | 1 | 0 | 1.1 | 12.2 | NA | NA | 288 | 0 | 0 | 0 | 0 | 0 | 0 | 0 | 0 |
| P494 | 1 | 92 | SJHC 1 EMERGENCY | 1 | 3 | 1600 | 2 | 0 | NA | NA | NA | NA | 150 | 0 | 0 | 0 | 0 | 0 | 0 | 0 | 0 |
| P84 | 1 | 72 | HGH EMERGENCY DEPT | 1 | 1 | 1670 | 2 | 0 | 1.1 | 13 | 26 | NA | 283 | 0 | 0 | 0 | 1 (US) | 0 | 0 | 0 | 0 |
| P108 | 1 | 49 | HGH EMERGENCY DEPT | 1 | 1 | 1610 | 2 | 0 | NA | NA | NA | NA | 38 | 0 | 0 | 0 | 1 (US & CTPA) | 1 | 0 | 1 | 0 |
| P280 | 0 | 31 | JH EMERGENCY DEPT | 1 | 2 | 1090 | 2 | 0 | 1.1 | 12.4 | NA | NA | 212 | 0 | 0 | 0 | 1 (CTPA) | 0 | 0 | 0 | 0 |
| P480 | 0 | 88 | SJHC 5MG CTU | 1 | 2 | 2150 | 2 | 0 | 1.1 | 13.2 | 27 | NA | 301 | 0 | 0 | 0 | 1 (US) | 0 | 1 | 0 | 0 |
| P105 | 1 | 78 | HGH EMERGENCY DEPT | 1 | 3 | 371 | 1 | 0 | 1.1 | 12.4 | NA | NA | 247 | 0 | 0 | 1 (NSTEMI) | 0 | 0 | 0 | 0 | 0 |
| P483 | 0 | 61 | SJHC 1 EMERGENCY | 1 | 3 | 4090 | 2 | 0 | 1.6 | 19.1 | 34 | 1.9 | 79 | 0 | 0 | 1 (Right iliac artery confirmed by CTA) | 1 (CTA, US, & CTPA) | 1 | 0 | 0 | 0 |
| P363 | 0 | 40 | MM WOMENS ICU | 1 | 4 | 1320 | 2 | 0 | 1.3 | 14.5 | 29 | 4.6 | 82 | 0 | 0 | 0 | 0 | 0 | 0 | 0 | 0 |
| P282 | 1 | 80 | JH EMERGENCY DEPT | 1 | 2 | 6160 | 2 | 0 | 1.2 | 13.9 | 31 | NA | 155 | 0 | 0 | 0 | 1 (CT of chest, abdomen, pelvis) | 0 | 0 | 0 | 1 |
| P495 | 0 | 25 | SJHC 1 EMERGENCY | 1 | 2 | 1650 | 2 | 0 | NA | NA | NA | NA | 235 | 0 | 0 | 0 | 1 (US & CTPA) | 0 | 0 | 0 | 0 |
| P496 | 1 | 80 | SJHC 5MG CTU | 1 | 2 | 3890 | 2 | 0 | NA | NA | NA | NA | 171 | 0 | 0 | 0 | 0 | 0 | 0 | 0 | 0 |
| P107 | 0 | 94 | HGH EMERGENCY DEPT | 1 | 1 | 1360 | 2 | 0 | 0.9 | 10.6 | 28 | NA | 166 | 0 | 0 | 0 | 1 (US) | 0 | 0 | 0 | 0 |
| P283 | 1 | 71 | JH EMERGENCY DEPT | 1 | 1 | 934 | 2 | 0 | 1 | 12.1 | 34 | NA | 209 | 0 | 0 | 0 | 1 (US) | 0 | 0 | 0 | 0 |
| P106 | 0 | 57 | HGH EMERGENCY DEPT | 1 | 3 | 307 | 1 | 0 | 0.9 | 10.6 | NA | NA | 492 | 0 | 0 | 1 (NSTEMI) | 0 | 0 | 0 | 0 | 0 |

APTT, activated partial thromboplastin time; COPD, chronic obstructive pulmonary disease; CTA, computed tomography angiography; CTPA, computed tomography pulmonary angiography; DIC, disseminated intravascular coagulopathy; DVT, deep venous thrombosis; HLH, hemophagocytic lymphohistocytosis; INR, international normalized ratio; ISTH, International Society on Thrombosis and Hemostasis; MRI, magnetic resonance imaging; NM VQ, nuclear medicine ventilation perfusion scan; NSTEMI, Non-ST- elevation myocardial infarction; PE, pulmonary embolism; PERC, pulmonary embolism rule-out criteria; PT, prothrombin time; STEMI, ST-segment elevation myocardial infarction; tNHL, transformed non-Hodgkin lymphoma; US, ultrasound; VTE, venous thromboembolism; XR, X-ray; YEARS, clinical decision rule for pulmonary embolism.

Collection departments abbreviated: 4W CARDIOLOGY, inpatient cardiology ward; 4W VASCULAR, vascular inpatient ward; 5MG CTU, inpatient internal medicine clinical teaching unit; 5W MEDICINE, inpatient medical ward; 6W ORTHO, inpatient orthopedic ward; 6S SURG/TRAUMA, inpatient surgery/trauma ward; 7S STROKE/NEURO, inpatient stroke/neurology ward; B4 HEMATOLOGY, hematology inpatient ward; C3 ONCOLOGY/GI, inpatient oncology/gastrointestinal unit; DEPT, department; E3 MEDICINE, inpatient medical unit; E4 SURGERY, inpatient surgery ward; GH EMERGENCY DEPT, Hamilton General emergency department; HGH; Hamilton General Hospital; ICU, intensive care un unit; JCC, Juravinski cancer center; JH, Juravinski Hospital; ODS, oncology day service; LAB SCC, outpatient laboratory; MM, McMaster University Medical Centre; SJHC 4D CTU, inpatient internal medicine clinical teaching unit; SJHC MSAU H&N, inpatient medical surgical assessment unit head and neck unit; SJHC MSAU SSDU, inpatient medical/surgical assessment unit; SJHC/ SJHK; St. Joseph’s Healthcare; T7 NEPHROLOGY, inpatient nephrology ward; WEC URGENT CARE CENTRE, West End Clinic urgent care centre (Hamilton Health Sciences).

**Table S3.** **Comparison of D-dimer levels among the consecutive adult and pediatric patients who had D-dimer tests for different reasons.**

|  | **Results for Children** | **Results for Adults** |
| --- | --- | --- |
| **Reason for D-dimer testing** | **% with elevated D-dimer**  **(median, range and IQR of elevated values in μg/L FEU)** | |
|  |  |  |
| **Assessment of possible VTE (Pediatric=78, Adults=139)**  Query PE (Pediatric=61, Adult=89), DVT (Pediatric=13, Adult=50) or other site thrombosis (Pediatric=4) | 33.3%  Median: 1010  Range: 522 to 31,100  IQR: 662-2690 | 65%  Median: 1,380  Range: 500 to 21,100  IQR: 887-2190 |
| **Assessment during anticoagulation for VTE (Pediatric=10, Adults=2)**  up to 10 days after a newly diagnosed VTE (Pediatric=8) or later (Pediatric=2, Adult= 2) | 80%  Median: 10,800  Range: 759 to 119,000  IQR: 1,060-25,600 | 50% |
| **Assessment for arterial thrombosis (Pediatric=8, Adult=42)**  Query stroke (Pediatric=5, Adult=2), cardiac (Adult=39), or extremity (Pediatric=3, Adult=1) arterial thrombosis | 62.5%  Median: 1,630  Range: 736 to 2020  IQR: 1,290-1,630 | 40%  Median: 1,210  Range: 534 to 1,4900  IQR: 870-2,330 |
| **Assessment for Inflammatory and/or infectious (n=40), including:**  Query KD or MIS-C (N=20) | 100%  Median: 1,620  Range: 694 to 6540  IQR: 1,020-2,120 |  |
| Confirmed or suspected COVID-19 (n=16) | 62.5%  Median: 1,190  Range: 889 to 16,200  IQR: 984-3,610 |  |
| sJIA (n=3) | 100%  Median: 5,260  Range: 3,590 to 24,000 |  |
| **Assessment during (CAR) T-cell therapy** (Pediatric=1, Adult=3) | 0% | 100%  Median: 3,660  Range: 1,670 to 3,870  IQR: 2,660-3,760 |
| Assessment for DIC (Pediatric n=31: systemic DIC n=27; localized from an AVM n=4; Adult n=9 systemic) | 71%  Median: 3,530  Range: 797 to >128,000  IQR: 1,670-11,200 | 82%  Median: 3,220  Range: 642 to 6,650  IQR: 1,230-5,390 |
| **Assessment for undetermined reasons (Pediatric=8, Adult=5)** | 25%  972 and 996 | 60%  Median: 1,030  Range: 860 to 4440  IQR: 950-2740 |

IQR: interquartile range; VTE: venous thromboembolism; PE: pulmonary embolism; DVT: deep venous thrombosis; KD: Kawasaki disease; MIS-C: multisystem inflammatory syndrome in children; sJIA: systemic juvenile idiopathic arthritis; (CAR): chimeric antigen receptor; DIC: disseminated intravascular coagulopathy; AVM: congenital arterial-venous malformation.

**Table S4. Summary of data for the adult and pediatric patients who had five or more D-dimer tests.** Information on ages, sex, diagnoses and laboratory results were obtained by review of electronic medical records. The reasons for D-dimer testing are coded as follows: 3, arterial thrombosis; 4, DIC; 5, chimeric antigen receptor T-cell therapy; 6, COVID-19; 7, inflammatory conditions (MIS-C/KD/sJIA); 8, other VTE exclusion; 9, other causes; 10, VTE assessment; 11, ECMO. Abbreviations for the diagnoses are shown below the table.

| Code | sex  0=F  1=M | age  (yrs) | collection  department | alive on discharge 0=no 1=yes | reason for D-dimer testing | Disease treated by CAR T-cell | first  D-dimer result  (µg/FEU) | D-dimer elevated 1=no,  2=yes | total #  of  D-dimer repeats | median  D-dimer result  (µg/FEU) | tested  for  how  long  (days) | tested more  than once a day  0=no 1=yes | maximum D-dimer result  (µg/L FEU) | Nadir D-dimer result  (µg/L FEU) | maximal difference in D-dimer per day  (µg/L FEU) | INR | PT  (sec) | APTT  (sec) | Fibrinogen (g/l) | lowest  platelet  count (x10^9^/L) | cause  of  DIC | one or more ISTH DIC scores recorded on chart 0=no  1=yes |
| --- | --- | --- | --- | --- | --- | --- | --- | --- | --- | --- | --- | --- | --- | --- | --- | --- | --- | --- | --- | --- | --- | --- |
| P2980 | 0 | 71 | JH ODS | 1 | 5 | DLBCL | <215 | 1 | 11 | 447 | 31 | 0 | 760 | 215 | NA | 1.6 | 19.2 | 45 | 2.2 | 52 | NA | NA |
| P1897 | 1 | 60 | JH ODS | 1 | 5 | MCL | 447 | 1 | 52 | 514 | 61 | 0 | 1870 | 215 | NA | 1.3 | 14.4 | 35 | 1.7 | 14 | NA | NA |
| P336 | 0 | 72 | JH ODS | 1 | 5 | DLBCL | 395 | 1 | 18 | 619 | 32 | 0 | 1810 | 383 | NA | 1.8 | 20.5 | 35 | 3.3 | 51 | NA | NA |
| P6096 | 1 | 72 | JH ODS | 1 | 5 | DLBCL | 648 | 2 | 19 | 640 | 34 | 0 | 1060 | 286 | NA | 1.1 | 12.6 | 31 | 3.4 | 121 | NA | NA |
| P6407 | 1 | 66 | JH ODS | 1 | 5 | MCL | 478 | 1 | 24 | 642 | 32 | 0 | 3790 | 236 | NA | 1.2 | 13.1 | 36 | 1.6 | 35 | NA | NA |
| P6435 | 1 | 70 | JH ODS | 1 | 5 | DLBCL | 472 | 1 | 22 | 712 | 34 | 0 | 1290 | 404 | NA | 1.5 | 17 | 31 | 1.4 | 26 | NA | NA |
| P2106 | 1 | 69 | JH ODS | 1 | 5 | DLBCL | 752 | 2 | 23 | 752 | 63 | 0 | 1090 | 248 | NA | 2.9 | 33.1 | 49 | 1.3 | 18 | NA | NA |
| P2990 | 1 | 62 | JH ODS | 1 | 5 | DLBCL | 1350 | 2 | 21 | 810 | 34 | 0 | 2010 | 550 | NA | 1.2 | 14.2 | 40 | 2.3 | 59 | NA | NA |
| P725 | 1 | 58 | JH ODS | 1 | 5 | DLBCL | 925 | 2 | 21 | 888 | 38 | 0 | 6020 | 357 | NA | 1.4 | 16.6 | 36 | 1.4 | 100 | NA | NA |
| P2464 | 1 | 54 | JH ODS | 1 | 5 | DLBCL | 1050 | 2 | 26 | 968 | 48 | 0 | 5320 | 370 | NA | 1.5 | 17 | 77 | 1.6 | 16 | NA | NA |
| P290 | 1 | 26 | JH ODS | 1 | 5 | DLBCL | 1060 | 2 | 18 | 1050 | 34 | 0 | 1750 | 756 | NA | 1.3 | 15.3 | 37 | 2.9 | 76 | NA | NA |
| P5040 | 1 | 45 | JH ODS | 1 | 5 | DLBCL | 1280 | 2 | 22 | 1060 | 35 | 0 | 7250 | 497 | NA | 2 | 22.6 | 102 | 0.8 | 55 | NA | NA |
| P298 | 0 | 72 | JH ODS | 1 | 5 | DLBCL | 1070 | 2 | 25 | 1070 | 47 | 0 | 3160 | 556 | NA | 1.9 | 22.6 | 30 | 1.5 | 44 | NA | NA |
| P7488 | 1 | 62 | JH ODS | 1 | 5 | DLBCL | 1170 | 2 | 20 | 1100 | 32 | 0 | 2480 | 620 | NA | 1.3 | 15.2 | 32 | 1.3 | 57 | NA | NA |
| P2978 | 1 | 65 | JH ODS | 1 | 5 | DLBCL | 1070 | 2 | 23 | 1120 | 40 | 0 | 6680 | 589 | NA | 1.5 | 18.3 | 33 | 1.3 | 67 | NA | NA |
| P3397 | 1 | 59 | JH ODS | 1 | 5 | DLBCL | 1860 | 2 | 53 | 1220 | 27 | 0 | 1570 | 588 | NA | 1.6 | 18.4 | 50 | 2.7 | 119 | NA | NA |
| P2553 | 1 | 60 | JH ODS | 1 | 5 | DLBCL | 2370 | 2 | 40 | 1230 | 48 | 1 | 12400 | 648 | 130 | 1.3 | 15 | 78 | 1.2 | 5 | NA | NA |
| P709 | 0 | 68 | JH ODS | 1 | 5 | MM | 348 | 1 | 22 | 1350 | 33 | 0 | 4760 | 707 | NA | 1.2 | 13.7 | 37 | 3.6 | 103 | NA | NA |
| P4963 | 0 | 64 | JH ODS | 1 | 5 | DLBCL | 800 | 2 | 19 | 1700 | 37 | 1 | 6500 | 800 | 169 | 1.3 | 15.1 | 37 | 2 | 46 | NA | NA |
| P2456 | 1 | 76 | JH ODS | 1 | 5 | MM | 1470 | 2 | 25 | 1740 | 42 | 0 | 34500 | 769 | NA | 1.2 | 13.6 | 36 | 1.3 | 29 | NA | NA |
| P4614 | 1 | 72 | JCC LAB SCC | 1 | 5 | DLBCL | 4030 | 2 | 28 | 2000 | 41 | 0 | 13500 | 913 | NA | 2.1 | 24.5 | 42 | 1.5 | 64 | NA | NA |
| P1153 | 0 | 63 | JH ODS | 1 | 5 | DLBCL | 1860 | 2 | 20 | 2460 | 38 | 0 | 7300 | 1130 | NA | 1.2 | 14 | 27 | 2.4 | 20 | NA | NA |
| P4970 | 1 | 59 | JH C4 HEMATOLOGY | 0 | 5 | DLBCL | 4070 | 2 | 8 | 2490 | 7 | 0 | 5490 | 1770 | NA | 1.7 | 19 | 87 | 2 | 82 | NA | NA |
| P741 | 1 | 58 | JH ODS | 0 | 5 | DLBCL | 3440 | 2 | 12 | 3250 | 32 | 0 | 4420 | 1560 | NA | 1.9 | 21.8 | 36 | 3.5 | 7 | NA | NA |
| P2555 | 1 | 52 | JH ODS | 1 | 5 | DHL | 5100 | 2 | 10 | 3300 | 34 | 0 | 5100 | 983 | NA | 1.2 | 14 | 30 | 1.3 | 67 | NA | NA |
| P216 | 1 | 67 | JH ODS | 1 | 5 | THL | 1070 | 2 | 28 | 3400 | 47 | 0 | 7100 | 1070 | NA | 1.3 | 15.5 | 28 | 2.7 | 5 | NA | NA |
| P7418 | 1 | 67 | JH ODS | 1 | 5 | MCL | 695 | 2 | 22 | 3590 | 25 | 0 | 5390 | 215 | NA | 2.3 | 26.8 | 43 | 1 | 8 | NA | NA |
| P2145 | 1 | 71 | JH ODS | 1 | 5 | DLBCL | 1630 | 2 | 30 | 3810 | 48 | 0 | 12100 | 861 | NA | 1.5 | 17.6 | 41 | 1.7 | 3 | NA | NA |
| P5330 | 0 | 65 | JH ODS | 0 | 5 (not done) | DLBCL | 1860 | 2 | 12 | 3840 | 25 | 0 | 5290 | 1540 | NA | 2.1 | 32.6 | 53 | 2.5 | 3 | NA | NA |
| P3741 | 0 | 73 | JH ODS | 1 | 5 | DLBCL | 1500 | 2 | 36 | 4330 | 49 | 0 | 15800 | 1180 | NA | 1.4 | 15.8 | 28 | 1.1 | 8 | NA | NA |
| P262 | 1 | 62 | JH ODS | 1 | 5 | DLBCL | 3860 | 2 | 32 | 4540 | 55 | 0 | 20000 | 1020 | NA | 1.2 | 13.8 | 26 | 1.2 | 77 | NA | NA |
| P294 | 1 | 45 | JH ICU LEVEL 0 | 0 | 5 | THRLBCL | 4730 | 2 | 29 | 5240 | 39 | 0 | 7500 | 2050 | NA | 2 | 23.7 | 32 | 0.7 | 5 | NA | NA |
| P5718 | 1 | 71 | JH ODS | 1 | 5 | MCL | 641 | 2 | 74 | 6300 | 96 | 0 | 26100 | 641 | NA | 1.3 | 15.1 | 47 | 1.1 | 12 | NA | NA |
| P1183 | 1 | 62 | JH ODS | 1 | 5 | MM | 6760 | 2 | 12 | 6310 | 14 | 1 | 7120 | 4510 | 189 | 1.2 | 13.7 | 34 | 2.9 | 119 | NA | NA |
| P2067 | 0 | 37 | JH ODS | 1 | 5 | DLBCL | 128000 | 2 | 31 | 6540 | 51 | 0 | 128000 | 776 | NA | 1.8 | 20.8 | 61 | 0.6 | 28 | NA | NA |
| P6492 | 1 | 63 | JH ODS | 1 | 5 | DLBCL | 4890 | 2 | 23 | 7420 | 26 | 0 | 18000 | 224 | NA | 1.3 | 14.8 | 30 | 1.5 | 17 | NA | NA |
| P1105 | 0 | 48 | HGH ICU EAST/SOUTH | 1 | 4 | NA | 1930 | 2 | 12 | 1930 | 13 | 0 | 4530 | 1380 | NA | 1.7 | 19.3 | 35 | 1.8 | 31 | sepsis | 0 |
| P1468 | 1 | 67 | HGH ICU EAST/SOUTH | 1 | 4 | NA | 89700 | 2 | 15 | 14600 | 21 | 1 | 89000 | 7350 | 262 | 2.3 | 26.5 | 64 | 2.9 | 30 | sepsis | 1 |
| P1514 | 0 | 25 | HGH 7W NEUROSURGERY | 1 | 4 | NA | 406 | 1 | 9 | 599 | 9 | 0 | 1880 | 406 | NA | 1.7 | 28.4 | 54 | 2 | 16 | sepsis | 0 |
| P190 | 1 | 75 | JH C4 HEMATOLOGY | 0 | 4 | NA | 2330 | 2 | 14 | 7280 | 16 | 0 | 16800 | 2330 | NA | 1.4 | 16.3 | 31 | 7.2 | 63 | post BM transplant | 0 |
| P2350 | 0 | 49 | HGH 8W MEDICINE | 0 | 4 | NA | 760 | 2 | 39 | 4660 | 47 | 0 | 17700 | 1020 | NA | 3.5 | 39.9 | 90 | 0.5 | 28 | sepsis | 1 |
| P2548 | 1 | 38 | JH ICU LEVEL 1 | 1 | 4 | NA | 4100 | 2 | 6 | 9430 | 6 | 1 | 18100 | 4100 | 396 | 1.8 | 15.5 | 49 | 1.8 | 31 | malignancy | 0 |
| P2773 | 1 | 85 | HGH EMERGENCY DEPT | 1 | 4 | NA | 17400 | 2 | 5 | 22800 | 8 | 0 | 31700 | 14500 | NA | 1.7 | 19.8 | 33 | 0.5 | 54 | malignancy | 0 |
| P2792 | 1 | 44 | HGH BURN UNIT | 1 | 4 | NA | 1620 | 2 | 7 | 2320 | 12 | 1 | 6750 | 1170 | 227 | 2.2 | 25.1 | 30 | 4.9 | 36 | necrotic tissue | 0 |
| P1645 | 0 | 68 | JH EMERGENCY DEPT | 1 | 4 | NA | 4830 | 2 | 7 | 2150 | 14 | 1 | 6750 | 1400 | 1920 | 2 | 24.1 | 30 | 4.4 | 503 | malignancy | 0 |
| P4032 | 1 | 55 | HGH CARD/VASC ICU | 1 | 4 | NA | 4300 | 2 | 15 | 12800 | 37 | 0 | 65800 | 2820 | NA | 8.4 | 99.6 | 42 | 2.8 | 61 | sepsis | 0 |
| P420 | 0 | 72 | HGH CARD/VASC ICU | 0 | 4 | NA | 4590 | 2 | 16 | 11200 | 40 | 0 | 15700 | 4120 | NA | 4.6 | 53.8 | 33 | 1.1 | 42 | sepsis | 0 |
| P4252 | 0 | 7 | MM PICU | 1 | 4 | NA | 12500 | 2 | 11 | 12500 | 11 | 1 | 19000 | 5400 | 2430 | 1.3 | 14.6 | 37 | 4.2 | 11 | sepsis | 0 |
| P4464 | 0 | 80 | HGH 6W ORTHO | 1 | 4 | NA | 1390 | 2 | 6 | 983 | 7 | 0 | 1390 | 528 | NA | 1 | 11.7 | 30 | 2.7 | 20 | unknown cause | 0 |
| P4549 | 0 | 77 | HGH EMERGENCY DEPT | 0 | 4 | NA | 7370 | 2 | 5 | 6210 | 7 | 0 | 7370 | 6000 | NA | 6.2 | 71.8 | >200 | 1.1 | 20 | unknown cause | 0 |
| P4581 | 1 | 31 | JH C4 HEMATOLOGY | 1 | 4 | NA | 6030 | 2 | 86 | 1170 | 216 | 0 | 70700 | 286 | NA | 1.6 | 19.3 | 51 | 3.1 | 7 | malignancy | 0 |
| P4629 | 1 | 63 | JH B4 HEMATOLOGY | 1 | 4 | NA | 28200 | 2 | 16 | 6920 | 6 | 1 | 28200 | 4690 | 5540 | 2.1 | 24.9 | 37 | 1.7 | 21 | malignancy | 0 |
| P4848 | 0 | 60 | HGH ICU EAST/SOUTH | 1 | 4 | NA | 18100 | 2 | 5 | 14300 | 6 | 0 | 18100 | 6350 | NA | 1.6 | 19 | 26 | 2.1 | 19 | sepsis | 0 |
| P4901 | 0 | 60 | HGH 7S STROKE/NEURO | 1 | 4 | NA | 2960 | 2 | 14 | 1170 | 28 | 0 | 2960 | 605 | NA | 1.2 | 13.7 | 125 | 2.4 | 37 | ?CAPS | 1 |
| P4931 | 1 | 50 | JH C4 HEMATOLOGY | 1 | 4 | NA | 79900 | 2 | 15 | 57200 | 4 | 1 | 85400 | 28300 | 19700 | 1.7 | 19.5 | 24 | 1.1 | 12 | malignancy | 0 |
| P7109 | 1 | 30 | JH ICU LEVEL 0 | 0 | 4 | NA | 6430 | 2 | 15 | 5850 | 26 | 0 | 34200 | 1730 | NA | 2.4 | 28.1 | 59 | 1.3 | 7 | malignancy | 0 |
| P4985 | 1 | 64 | HGH EMERGENCY DEPT | 1 | 4 | NA | 13300 | 2 | 7 | 6780 | 8 | 1 | 13300 | 6120 | 5840 | 1 | 11.6 | 26 | 1.3 | 51 | unknown cause | 1 |
| P5 | 1 | 29 | HGH 8W MEDICINE | 1 | 4 | NA | 6200 | 2 | 23 | 11900 | 38 | 0 | 24100 | 4480 | NA | 1 | 11.4 | 26 | 2.2 | 39 | unknown cause | 0 |
| P5213 | 1 | 68 | HGH ICU WEST | 1 | 4 | NA | 6060 | 2 | 20 | 5020 | 21 | 0 | 25800 | 3610 | NA | 4.5 | 52.5 | 38 | 5.2 | 17 | sepsis | 0 |
| P5253 | 1 | 85 | HGH CARD CARE UNIT | 0 | 4 | NA | 33300 | 2 | 6 | 7270 | 8 | 0 | 33300 | 3710 | NA | 2 | 22.1 | 40 | 1.7 | 37 | malignancy | 0 |
| P5522 | 0 | 73 | SJHC 1 EMERGENCY | 1 | 4 | NA | 4470 | 2 | 6 | 21700 | 7 | 0 | 54500 | 4470 | NA | 1.7 | 19.1 | 28 | 2.7 | 40 | malignancy | 0 |
| P5712 | 1 | 24 | JH C4 HEMATOLOGY | 1 | 4 | NA | 2120 | 2 | 35 | 3860 | 83 | 1 | 30700 | 1650 | 9140 | 1.4 | 16.1 | 39 | 1.1 | 6 | malignancy | 0 |
| P5931 | 0 | 77 | HGH EMERGENCY DEPT | 1 | 4 | NA | 63500 | 2 | 14 | 14040 | 14 | 0 | 63500 | 4600 | NA | 1.7 | 19.1 | >200 | 1.2 | 39 | unknown cause | 0 |
| P5953 | 0 | 71 | HGH ICU EAST/SOUTH | 0 | 4 | NA | 6780 | 2 | 5 | 6270 | 4 | 1 | 7500 | 5890 | 652 | >10 | >130 | >200 | <0.4 | 35 | sepsis | 1 |
| P6302 | 1 | 60 | HGH ICU EAST/SOUTH | 1 | 4 | NA | 38700 | 2 | 5 | 4560 | 5 | 0 | 38700 | 2510 | NA | 2.9 | 32.8 | >200 | 0.7 | 103 | drowning post cardiac arrest | 0 |
| P6354 | 1 | 49 | HGH CARD CARE UNIT | 1 | 4 | NA | 1070 | 2 | 6 | 425 | 6 | 0 | 1070 | 323 | NA | 2.2 | 25.3 | 79 | 3.3 | 50 | CAPS | 0 |
| P6478 | 0 | 22 | JH B4 HEMATOLOGY | 1 | 4 | NA | 29400 | 2 | 7 | 128000 | 7 | 0 | 128000 | 29400 | NA | 2.6 | 30.5 | 48 | 1 | 18 | malignancy | 0 |
| P6824 | 1 | 71 | JH ODS | 1 | 4 | NA | 1140 | 2 | 5 | 1340 | 32 | 0 | 1530 | 1130 | NA | 1.1 | 12.7 | 29 | 1.1 | 17 | post SCT | 0 |
| P702 | 0 | 53 | JH C4 HEMATOLOGY | 1 | 4 | NA | 128000 | 2 | 5 | 128000 | 3 | 1 | 128000 | 93900 | 7810 | 1.3 | 15.7 | 25 | 1 | 27 | malignancy | 0 |
| P7646 | 0 | 64 | HGH ICU EAST/SOUTH | 0 | 4 | NA | 128000 | 2 | 25 | 5840 | 25 | 0 | 128000 | 1120 | NA | 2.6 | 29.7 | > 200 | 1.2 | 28 | sepsis | 1 |
| P7988 | 1 | 76 | SJHC 4 MED STEP DOWN | 1 | 4 | NA | 40100 | 2 | 8 | 5310 | 8 | 0 | 40100 | 1400 | NA | 1.4 | 16.1 | 33 | 1 | 13 | unknown cause | 1 |
| P8009 | 1 | 80 | HGH NEURO/TRAUMA ICU | 1 | 4 | NA | 825 | 2 | 20 | 4300 | 19 | 0 | 10700 | 1900 | NA | 1.7 | 18.8 | 45 | 1.3 | 18 | post trauma | 0 |
| P8023 | 1 | 78 | HGH CARD CARE UNIT | 1 | 4 | NA | 4060 | 2 | 9 | 4060 | 11 | 0 | 5240 | 2360 | NA | 2 | 22.4 | 45 | 1 | 36 | sepsis | 0 |
| P8330 | 1 | 61 | HGH CARD/VASC ICU | 1 | 4 | NA | 14900 | 2 | 12 | 14500 | 22 | 0 | 28600 | 7200 | NA | 7.3 | 86.3 | 30 | < 0.4 | 29 | post-surgery | 0 |
| P8392 | 0 | 66 | JH EMERGENCY DEPT | 1 | 4 (TTP) | NA | 5320 | 2 | 35 | 12600 | 31 | 1 | 35000 | 2640 | 2180 | 1.1 | 12.3 | 32 | 2.8 | 8 | TTP | 0 |
| P8749 | 0 | 21 | MM WOMENS ICU | 1 | 4 | NA | 128000 | 2 | 7 | 5700 | 6 | 1 | 128000 | 1160 | 38100 | 1.5 | 17.2 | NA | 0.8 | 116 | sepsis | 0 |
| P9127 | 1 | 55 | HGH MED/SURG ICU | 0 | 4 | NA | 128000 | 2 | 7 | 18200 | 17 | 0 | 128000 | 10800 | NA | 2 | 22.2 | 86 | 3.9 | 33 | sepsis | 0 |
| P996 | 0 | 70 | HGH 8W MEDICINE | 1 | 4 | NA | 722 | 2 | 18 | 1240 | 20 | 0 | 3210 | 675 | NA | 3.3 | 32.6 | 50 | 0.6 | 56 | sepsis | 1 |
| P3282 | 1 | 36 | HGH ICU EAST/SOUTH | 1 | 11 | NA | 3070 | 2 | 15 | 3070 | 15 | 0 | 11300 | 768 | NA | 1 | 11.6 | 46 | 2.8 | 98 | NA | NA |
| P1506 | 1 | 62 | HGH MAIN OR | 0 | 11 | NA | 711 | 2 | 9 | 11100 | 8 | 1 | 55300 | 644 | 204 | 1.7 | 19.4 | 97 | 1.6 | 63 | NA | NA |
| P4909 | 1 | 53 | HGH ICU WEST | 1 | 11 | NA | 1080 | 2 | 7 | 1830 | 7 | 0 | 10900 | 858 | NA | 1.2 | 14.4 | 69 | 3 | 111 | NA | NA |
| P1452 | 1 | 45 | HGH ICU WEST | 1 | 11 | NA | 7270 | 2 | 6 | 18100 | 6 | 0 | 101000 | 1440 | NA | 3.3 | 37.9 | >200 | 1.3 | 25 | NA | NA |
| P5230 | 1 | 55 | HGH ICU WEST | 1 | 11 | NA | 506 | 2 | 6 | 735 | 6 | 0 | 1340 | 476 | NA | 2.4 | 27.1 | 104 | 2 | 49 | NA | NA |
| P5992 | 1 | 58 | HGH ICU WEST | 1 | 11 | NA | 1550 | 2 | 5 | 1550 | 5 | 0 | 6160 | 1070 | NA | 1.4 | 16 | 140 | 3 | 72 | NA | NA |
| P2762 | 0 | 30 | HGH EMERGENCY DEPT | 0 | 11 | NA | 449 | 1 | 5 | 7470 | 6 | 0 | 50200 | 449 | NA | 2.3 | 26.7 | >200 | 1.2 | 39 | NA | NA |
| P7066 | 1 | 58 | HGH EMERGENCY DEPT | 1 | 11 | NA | 1170 | 2 | 10 | 15300 | 20 | 1 | 28200 | 1170 | 4120 | 2.5 | 28.6 | 102 | 2 | 40 | NA | NA |
| P2850 | 1 | 78 | HGH EMERGENCY DEPT | 1 | 6 | NA | 7210 | 2 | 20 | 12400 | 27 | 0 | 30200 | 5260 | NA | 2.9 | 32.9 | 37 | 1.5 | 54 | NA | NA |
| P1292 | 0 | 5 | MM PICU | 1 | 6 | NA | 889 | 2 | 5 | 370 | 5 | 0 | 1250 | 229 | NA | 1.7 | 19.2 | 37 | 2 | 216 | NA | NA |
| P3423 | 1 | 8 | MM EMERGENCY DEPT | 1 | 6 | NA | 256 | 1 | 5 | 327 | 8 | 0 | 578 | 256 | NA | 1.1 | 13.2 | 30 | 2.1 | 46 | NA | NA |
| P360 | 0 | 0 | MM EMERGENCY DEPT | 1 | 7 | NA | 3520 | 2 | 15 | 2840 | 13 | 1 | 3990 | 1230 | 1960 | 1.6 | 18.4 | 34 | 1.4 | 3 | NA | NA |
| P4678 | 1 | 3 | MM EMERGENCY DEPT | 1 | 7 | NA | 820 | 2 | 6 | 1310 | 10 | 0 | 1760 | 820 | NA | 1.6 | 18.9 | 37 | 7.6 | 224 | NA | NA |
| P1719 | 0 | 3 | MM PICU | 1 | 7 | NA | 4790 | 2 | 5 | 4050 | 7 | 0 | 4790 | 3240 | NA | 1.1 | 12.8 | 34 | 4 | 92 | NA | NA |
| P2167 | 0 | 15 | MM 3C MEDICINE | 1 | 7 | NA | 5260 | 2 | 6 | 1520 | 26 | 0 | 5260 | 630 | NA | 1.4 | 15.7 | 31 | 3.6 | 113 | NA | NA |
| P180 | 0 | 31 | HGH EMERGENCY DEPT | 1 | 10 | NA | 1280 | 2 | 8 | 9260 | 8 | 0 | 33700 | 1280 | NA | 1.7 | 18.6 | 36 | 1.5 | 16 | NA | NA |
| P352 | 0 | 13 | MM 3Z2 MEDICINE | 1 | 10 | NA | 119000 | 2 | 9 | 14500 | 49 | 0 | 119000 | 707 | NA | 1.1 | 13.3 | 33 | 7.4 | 478 | NA | NA |
| P6709 | 1 | 67 | HGH ICU EAST/SOUTH | 0 | 3 | NA | 6640 | 2 | 10 | 9600 | 10 | 0 | 25300 | 6160 | NA | 1.9 | 21.7 | 32 | 1.8 | 34 | NA | NA |
| P6545 | 1 | 4 | MM PICU | 1 | 3 | NA | 1630 | 2 | 21 | 2110 | 15 | 1 | 7180 | 591 | 956 | 1.3 | 14.4 | 30 | 2.4 | 117 | NA | NA |
| P5412 | 1 | 0 | MM NICU | 1 | 3 | NA | 2020 | 2 | 10 | 5260 | 9 | 1 | 19700 | 1040 | 5860 | 1.6 | 18.7 | 121 | 2.7 | 155 | NA | NA |
| P554 | 1 | 79 | HGH 4W CARDIOLOGY | 1 | 3 | NA | 6690 | 2 | 8 | 3910 | 9 | 0 | 6690 | 2270 | NA | 1 | 11.4 | 42 | 1.9 | 44 | NA | NA |

BM, bone marrow; CAPS, catastrophic antiphospholipid syndrome; DHL, double hit lymphoma; DLBCL, diffuse large B-cell lymphoma; MCL, mantel cell lymphoma; MM, multiple myeloma; NA, not applicable; SCT, stem cell transplant; THL, triple hit lymphoma; THRLBCL, T-cell histocyte rich large B-cell lymphoma; TTP, thrombotic thrombocytopenia purpura; VIIT, vaccine induced immune thrombocytopenia.

Collection department abbreviated: 3Z2 MEDICINE, pediatric inpatient medical unit; 4W CARDIOLOGY; 6W ORTHO, inpatient orthopedic unit; 7S STROKE/NEURO, inpatient stroke/neurology unit; 7W NEUROSURGERY, inpatient neurosurgery unit; 8W MEDICINE, inpatient medical unit; B4 hematology, inpatient hematology unit; C4 HEMATOLOGY, inpatient hematology unit; CARD CARE UNIT, inpatient cardiology care unit; CARD/VASC ICU, cardiology/vascular intensive care unit; DEPT, department; HGH, Hamilton General Hospital; ICU LEVEL 0, intensive care unit; ICU EAST/SOUTH, intensive care unit; JCC LAB SCC, Juravinski Hospital outpatient laboratory; JH, Juravinski Hospital; MED/SURG ICU, medical/surgical intensive care unit; MM, McMaster University Medical Centre; NICU, neonatal intensive care unit; ODS,oncology outpatient day service; OR, operation room; PICU, pediatric intensive care unit; SJHC, St. Joseph’s Healthcare.

**Table S5: Details of D-dimer levels, according to the reasons for D-dimer tests, among the 99 patients with ≥5 D-dimer tests.**

|  | Values as median, range, IQR | | | | | | |
| --- | --- | --- | --- | --- | --- | --- | --- |
| Reasons for multiple D-dimer testing ≥5 times | **Number of D-dimer tests** | **Duration of D-dimer monitoring, in days** | **Proportion with more than one D-dimer on any day** | **Maximal differences within one day between D-dimer levels**  **μg/L FEU** | **Initial D-dimer**  **μg/L FEU** | **Maximum D-dimer**  **μg/L FEU** | **Nadir D-dimer**  **μg/L FEU** |
| DIC (n=42, 41/42 adults, 1 child) | Median: 10  Range: 5 to 86  IQR: 6-16 | Median: 13  Range: 3 to 216  IQR: 7-24 | 30% | Median: 3,840  Range: 131 to 38,100  IQR: 819-7,810 | Median: 6,320  Range: 406 to >128,000  IQR: 2,490-32,300 | Median: 24,900  Range: 1,070 to >128,000  IQR: 7,400-50,900 | Median: 2,730  Range: 286 to 93,900  IQR: 1,230-5,760 |
| CAR T-cell therapy (n=36*, all adults) | Median: 23  Range: 8 to 74  IQR: 19-28 | Median: 36  Range: 7 to 96  IQR: 32-47 | 8.3% | Median: 169  Range: 130 to 189 | Median: 1,120  Range: <215 to 6,760  IQR: 693-2,050 | Median: 5,440  Range: 760 to >128,000  IQR: 2,360-8,640 | Median: 678  Range: <215 to 4,510  IQR: 399-1,040 |
| ECMO (n=8, all adults) | Median: 6.5  Range: 5 to 15  IQR: 6-9 | Median: 6.5  Range: 5 to 20  IQR: 6-10 | 25% | Median: 2,163  Range: 204 to 4,122  IQR: 1,180 to 3,140 | Median: 1,122.5  Range: 449 to 7,268  IQR: 658-1,930 | Median: 19,768  Range: 1,335 to 101,000  IQR: 9,730-51,500 | Median: 813  Range: 449 to 1,440  IQR: 602-1,091 |
| COVID-19 (n=3, 1 adult, 2 children) | Median: 5  Range: 5 to 20  IQR: 5-13 | Median: 8  Range: 5 to 27  IQR: 7-18 | 0 | 0 | Median: 889  Range: 256 to 7,209  IQR: 573-4,050 | Median: 1,250  Range: 578 to 30,200  IQR: 914-15,700 | Median: 256  Range: 229 to 5,260  IQR: 243-2,760 |
| Inflammatory Condition (MIS-C/KD/sJIA) (n=4, all children) | Median: 6  Range: 5 to 15  IQR: 6-8 | Median: 11.5  Range: 7 to 26  IQR: 9-16 | 25% | Only one time | Median: 4,154  Range: 820 to 5,260  IQR: 2,850-4,910 | Median: 4,391  Range: 1,760 to 5,260  IQR: 3,440-4,910 | Median: 1,024  Range: 630 to 3,240  IQR: 773-1,730 |
| VTE assessment (n=2, 1 adult and 1 child) | Median: 8.5  Range: 8 to 9  IQR: 8.25-8.75 | Median: 28.5  Range: 8 to 49  IQR: 18.25-38.75 | 0 | 0 | Median: 60,000  Range: 1,280 to 119,000  IQR: 30,600-89,400 | Median: 76,200  Range: 33,700 to 119,000  IQR: 54,900-97,500 | Median: 996  Range: 707 to 1,280  IQR: 851-1,140 |
| Arterial Thrombosis (n=4, 2/4 adult) | Median: 10  Range: 8 to 21  IQR: 10-13 | Median: 10  Range: 9 to 15  IQR: 9-11 | 50% | Median: 3,410  Range: 956 to 5,860  IQR: 2,180 to 4,630 | Median: 4,330  Range: 1,630 to 6,690  IQR: 1,920-6,650 | Median: 13,420  Range: 6,690 to 25,310  IQR: 7,060-21,100 | Median: 1,650  Range: 591 to 6,160  IQR: 925-3,240 |

DIC: disseminated intravascular coagulopathy; CAR: chimeric antigen receptor; ECMO: extracorporeal membrane oxygenation; MIS-C/KD/sJIA: Multi-system Inflammatory Syndrome in Children/Kawasaki disease/Systemic Juvenile Idiopathic Arthritis; VTE: venous thromboembolism; CNS: central nervous system; IQR: interquartile range; FEU: fibrinogen equivalent unit

*One patient who had started D-dimer level monitoring for CAR T-cell therapy died before receiving the CAR T-cell infusion
